# Supramolecular Assembly of Lanthanide-Binding Tag Peptides for Aqueous Separation of Rare Earth Elements

**DOI:** 10.1021/acsnano.5c05056

**Published:** 2025-10-06

**Authors:** Luis E. Ortuno Macias, Felipe Jiménez-Ángeles, Surabh S. KT, Kathleen J. Stebe, Monica Olvera de la Cruz, Mrinal K. Bera, Wei Bu, Binhua Lin, Charles Maldarelli, Raymond S. Tu

**Affiliations:** † Department of Chemical Engineering, 14770 The City College of New York, New York, New York 10031, United States; ‡ Levich Institute, The City College of New York, New York, New York 10031, United States; § Department of Materials Science and Engineering, 3270 Northwestern University, Evanston, Illinois 60208, United States; ∥ Department of Chemical and Biomolecular Engineering, 6572 University of Pennsylvania, Philadelphia, Pennsylvania 19104, United States; ⊥ NSF’s ChemMatCARS, Pritzker School of Molecular Engineering, 2462 University of Chicago, Chicago, Illinois 60637, United States

**Keywords:** rare earth elements, LBT peptides, separation, aggregation, condensation

## Abstract

Selective and eco-friendly separation and purification methods for rare earth elements (REEs) are necessary to meet the increasing demand for these valuable metals, which are extensively used in modern electronics and clean energy technologies. Mining feedstocks consist of REE mixtures as stable trivalent cations (Ln^3+^) that are difficult to separate due to their identical charge and similar size. Lanthanide-binding tags (LBTs), peptide chelates that coordinate Ln^3+^ in binding pockets, show promise as selective, high-affinity extractants. We demonstrate that the LBT variant LBTLLA^5–^, designed for high selectivity for Tb^3+^, is an effective extractant, forming complexes with REEs in solution that subsequently organize into self-assembling structures rich in Ln^3+^. These structures condense into aggregates that can be separated, enabling an efficient, all-aqueous, eco-friendly separation process. The self-assembled structures are studied using dynamic light scattering, ζ-potential measurements, transmission electron microscopy, anomalous small-angle X-ray scattering, inductively coupled plasma optical emission spectroscopy, and ultraviolet–visible absorption spectroscopy, which confirm LBTLLA^5–^ peptide-REE ion binding and the further assembly of micron-scale structures rich in REEs. Molecular dynamics simulations reveal the interactions promoting aggregation as well as the integrity of the binding pocket upon self-assembly. We find that LBTLLA^5–^:Ln^3+^ complexes recruit excess cations within the macrostructures, and we demonstrate that aggregation and selective separation can be controlled by manipulating the metal-peptide ratio in solution. Furthermore, we demonstrate separation from equimolar mixtures of REE pairs Tb^3+^-Lu^3+^ and Tb^3+^-La^3+^, supporting the application of LBT peptides as a platform for the selective separation of REEs.

## Graphical abstract

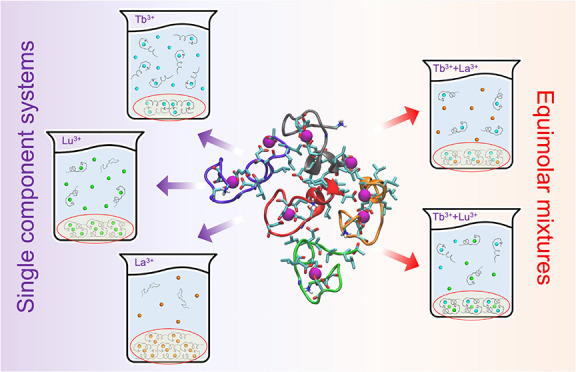

Advances to enable the selective separation of rare earth elements (REEs)—the metals lanthanum (La) through lutetium (Lu) in the periodic table, along with yttrium (Y) and scandium (Sc)—are now critically important as REEs with their luminescent, magnetic, and catalytic properties are essential materials in many modern technologies.^1–4^ Importantly, REEs are critical to a number of rapidly evolving clean and sustainable energy technologies including rechargeable batteries, wind turbines, solar panels, and hybrid vehicles.^5–8^ To date, REEs are primarily resourced through the mining of carbamate or phosphate ores or clays hosting these elements.^9^ Acid leaching of these ores or ion exchange with clays liberates the REEs from the solids as aqueous concentrate mixtures of trivalent lanthanum cations (Ln^3+^), and selective separation is required because the minerals contain more than one REE. This separation is challenging because of the identical charge of the Ln^3+^ cations and a similarity in their physicochemical properties.

Several chemical separation techniques are used to isolate individual REEs from mixed solutions, including ion exchange, chromatography, and solvent extraction.^10–13^ Among these, solvent extraction is the most widely used in commercial REE purification due to its operational simplicity, efficiency, and ability to handle large volumes.^13,14^ However, solvent extraction has considerable drawbacks, such as high energy consumption, pollution, and excessive solvent use due to the high viscosity of extractants employed in metal purification.^13,15,16^ These issues drive the need for alternative, environmentally sustainable separation methods. One promising alternative is solid-phase extraction (SPE),^17^ in which ligands (typically carboxylates and phosphates) are tethered onto surfaces, e.g., membranes,^18^ particles,^19^ microbe cells,^20–25^ or mesoporous materials (ion exchange resins,^26^ graphene oxide,^27^ silicas,^28^ nanogels,^29^ or metal–organic frameworks (MOFs)^30,31^ ). These approaches have the disadvantage that they offer poor selectivity among lanthanides, elevated consumption of energy, excessive production costs, and low capacity and require acidic conditions.

Biobased extraction platforms using peptides or proteins that selectively bind lanthanide cations^32–36^ have emerged as an environmentally sustainable alternative. Lanthanide-binding tags (LBTs) are short peptides (17–20 amino acids) that selectively coordinate Ln^3+^ by using loops that engage acidic residues and backbone carbonyl groups. Originally developed by Imperiali et al.^37–39^ from calcium-binding proteins, LBTs have been engineered for high-affinity binding to Tb^3+^, with sequences optimized through combinatorial peptide synthesis. The optimized LBT sequence, YIDTNNDGWYEGDELLA, exhibits a nanomolar dissociation constant and enhanced selectivity for Tb^3+^ over those of other lanthanides.

LBTs have been explored for REE separation. Park and Jiao et al.^40–43^ displayed LBT peptides on microbial cell surfaces, enabling selective REE binding through suspension, incubation, and centrifugation. Xie et al.^44^ attached LBT-modified microbes to silica particles, forming packed beds for REE separation. Renner et al.^45^ tethered peptide loops derived from lanthanide-binding proteins (e.g., Lanmodulin) to gold nanoparticles, using centrifugation for separation. Duval et al.^46^ functionalized a Lanmodulin-derived peptide on membranes platform for the adsorption of REEs commonly found in phosphogypsum waste streams. Lanthanide-binding peptides have also been immobilized on resin microbeads to selectively adsorb Tb^3+^ and Eu^3+^ from solution.^47^ Another study conjugated the EF-hand binding loop from Calmodulin to polymer scaffolds for cerium recovery.^48^

This study focuses on leveraging LBT self-assembly and aggregation for REE separation. When an LBT selectively binds a target Ln^3+^ in a mixed REE solution, aggregation conditions can induce precipitation of LBT:Ln^3+^ complexes, facilitating separation. Peptides and proteins naturally self-assemble into supramolecular structures via hydrophobic and electrostatic interactions.^49–52^ These interactions govern higher-order assembly, allowing biomolecules to recognize and organize around target ions.^53–56^ In prior work, we studied the adsorption of LBT:Ln^3+^ complexes to air-water interfaces for selective extraction. We demonstrated a modification of Imperiali et al.’s LBT optimized for Tb^3+^ coordination, denoted here as LBTLLA^5–^, with a hydrophobic sequence (LLA) addended at the C-terminus (i.e., YIDTNNDGWYEGDELLALLA) retains a binding selectivity for Tb^3+^,^57^ Figure 1A, and importantly greater surface activity than other LBT mutants because of the extended hydrophobic block (LLALLA), making it a good candidate for aggregation. Note that LBTLLA^5–^:Tb^3+^ association, as depicted in Figure 1B, results in a negatively charged complex (net charge of −2), with negative charge from carboxylate groups from the residue D11 and the C-terminus of the peptide (yellow circled groups in Figure 1B) and thus bridging of complexed LBTs with excess Ln^3+^ cations allows for a second driving force for aggregation.

**Figure 1:**
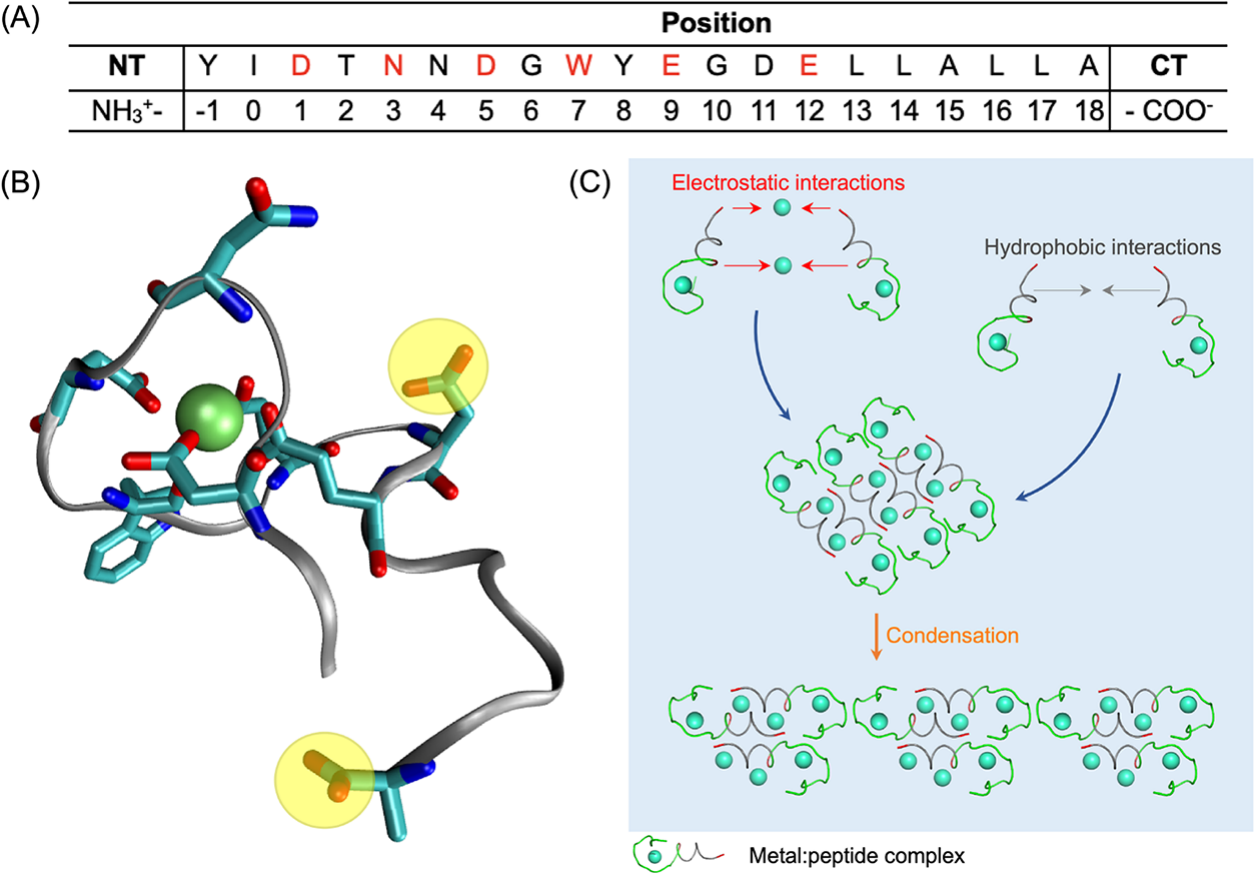
(A) Sequence alignment of LBTLLA^5–^ with amino acids coordinating with the Tb^3+^ colored in red, based on the crystallographic structure of the original mutant.^39^ NT and CT are the N-terminus and C-terminus, respectively, with charges corresponding to pH 6. (B) Molecular dynamics (MD) simulations snapshot of one of the stable peptide-Tb^3+^ conformations^57^ with carboxylate groups from D11 and the C-terminus highlighted in yellow. (C) Schematic representation of hypothesized self-assembly of metal-peptide complexes in solution and further condensation into aggregates. Carboxylate groups from D11 and C-terminus are colored in red, and hydrophobic block is colored in gray in the metal-peptide complexes represented in (C).

Here, we exploit the high affinity of the LBT peptide LBTLLA^5–^ with Tb^3+^ cations, as well as its hydrophobicity and surface charge to promote the supramolecular self-assembly of LBTLLA^5–^:Ln^3+^ complexes for the capture and selective separation of REEs. A peptide coordinating with one lanthanide ion can be associated by electrostatic interactions between LBTLLA^5–^:Ln^3+^ and free Ln^3+^ ions in solutions containing excess Ln^3+^. Additionally, the peptide’s hydrophobic properties enable the self-assembly of complexes through hydrophobic interactions (Figure 1C). By controlling the self-assembly of structures and promoting spontaneous precipitation, REEs can be effectively separated in an all-aqueous, environmentally friendly, and cost-effective separation process (Figure 1C).

The resulting peptide-metal structures are characterized using dynamic light scattering (DLS), ζ-potential measurements, and transmission electron microscopy (TEM). Detailed characterization of the lanthanide distribution within the self-assembled structures is achieved using anomalous small-angle X-ray scattering (ASAXS). Apart from measuring the size and morphology of the precipitates, ASAXS directly measures the number of ions per peptide and quantifies the selectivity of binding between terbium and lutetium. These measurements are corroborated by inductively coupled plasma optical emission spectroscopy (ICP-OES) and ultraviolet–visible (UV–vis) absorption spectroscopy. Additionally, molecular dynamics simulations provide insights into the factors driving aggregation and the stability of the binding pocket in the self-assembled structures.

Size and morphological characterization reveal that LBTLLA^5–^ aggregates form in the presence of excess ions without the need for an external energy input. We demonstrate that lanthanide cations are incorporated within the self-assembled structures, with a cation-to-peptide ratio greater than 1, indicating nonspecific electrostatic binding. Competitive binding studies with equimolar mixtures of Tb^3+^ and Lu^3+^, as well as Tb^3+^ and La^3+^, show selective separation patterns that differ from those observed under diluted conditions, where association constants are measured. Experiments show that the condensation of self-assembly structures depends on the type of lanthanide, the peptide complexes, and its concentration in solution. This dependency can be exploited as an environmentally viable alternative for the selective capture and separation of REEs.

## Results and Discussion

### Metal-Triggered Formation of Self-Assembly Peptide Fibrils

Supramolecular structure formation with the addition of Tb^3+^ cations in solution was monitored with DLS. Figure 2A shows the hydrodynamic diameter from the number-weighted distribution of species in solutions containing 100 μM LBTLLA^5–^ peptide and different concentrations of Tb^3+^ cations. These values are derived from the number-weighted distributions (reported in Figure S1), which are obtained from the intensity distribution using Mie theory. The error bars reported represent the width of the peaks from the number-based distribution (standard deviation), indicating the distribution of the peak (Figure S1). For Tb^3+^ cation concentrations of 0, 25, and 100 μM, which represent ratios of Tb^3+^ to peptide of 0, 0.25, and 1, respectively, the distribution indicates the presence of structures with a hydrodynamic diameter of approximately 0.92 nm. Therefore, these small structures indicate the existence of LBT peptide monomers and LBTLLA^5–^:Tb^3+^ complexes that are not assembled into larger superstructures, based on the dimensions of the simulated structure.^57^ Moreover, for Tb^3+^ concentrations of 400 μM and higher, the number-weighted intensity peak is shifted to a larger average size, with hydrodynamic diameter around 400 nm (see Figure 2A), indicating the existence of structures comprised of several individual LBTLLA^5–^:Tb^3+^ complexes. Although what triggers the formation of greater structures is not clear at this point, these results suggest that excess Tb^3+^ is needed for assembly at these concentrations. Furthermore, self-assembling structures are observed, particularly when the concentration of the trivalent cation exceeds the concentration of peptide in solution, suggesting that free Tb^3+^ in solution might induce the structural organization of the supramolecular cation-peptide complexes.

**Figure 2:**
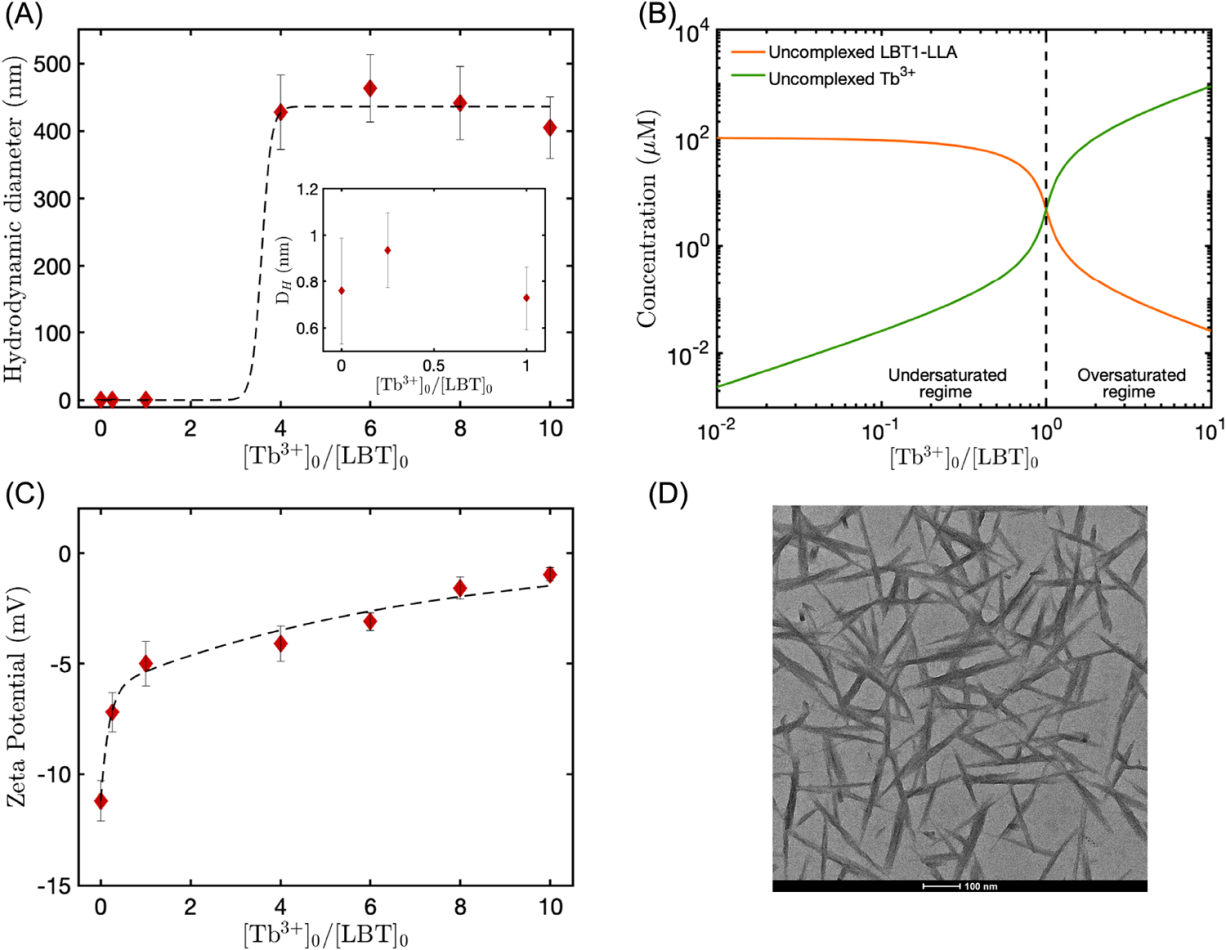
(A) Hydrodynamic diameter from the number-weighted intensity of species from solutions containing 100 μM LBTLLA^5–^ and different concentrations of Tb^3+^. The data were modeled using a sigmoidal function, represented by the dashed curve, to enhance visualization. (B) Calculated concentration of uncomplexed LBTLLA^5–^ and uncomplexed Tb^3+^ as a function of added Tb^3+^/LBTLLA^5–^ for a fixed LBT concentration of 100 μM and different Tb^3+^ concentrations. (C) ζ-Potential as a function of added Tb^3+^/LBTLLA^5–^ for a fixed LBT concentration of 100 μM and different Tb^3+^ concentrations. The data were modeled using an exponential function, represented by the dashed curve, to enhance visualization. (D) Dry TEM image from a solution containing 100 μM peptide and 400 μM Tb^3+^; similar structures were observed for Tb^3+^ concentrations higher than 400 μM. Error bars in panels (A) and (C) represent the standard deviation from three independent measurements.

Figure 2B shows the computed concentrations of uncomplexed peptide and unbound Tb^3+^ as a function of added terbium-to-peptide ratio for a fixed peptide concentration of 100 μM. These values are calculated based on the constant affinity of the peptide with Tb^3+^ cations,^57^ as detailed in the Supporting Information. Two well-defined regions can be observed in the figure: an undersaturated regime with Tb^3+^ concentrations lower than the concentration of peptide (100 μM) and an oversaturated regime with ratios of terbium to peptide higher than 1. Moreover, Figure 2B shows that the concentration of uncomplexed peptide decreases as the concentration of cations increases until it reaches a value close to 5 μM (quasi-saturation). After this point, the concentration of unbound peptide decreases slowly, to values close to zero. On the other hand, while the concentration of uncomplexed Tb^3+^ is not significant (gradually increases to values near 5 μM) for the undersaturated zone, it increases considerably for the saturated region as the density of cations increases in solution. Therefore, the monomeric and supramolecular states dependent on the Tb^3+^/LBT ratios observed in Figure 2A can be associated with the under- and oversaturated regimes. Ultimately, solutions containing monomeric species are in the undersaturated regime, while solutions containing supramolecular structures are in the oversaturated regime.

Surface charges of species in solutions were evaluated by measuring the ζ-potential of solutions containing 100 μM LBTLLA^5–^peptide and different concentrations of Tb^3+^ cations, corresponding to the under- and oversaturated regimes. Figure 2C shows that the unbound peptide possesses a negative surface charge as it is expected based on the negatively charged groups from the side chains of LBTLLA^5–^ at a pH of 6. As the Tb^3+^/LBT ratios increase, the ζ-potential becomes less negative, which was also expected since the coordination of LBT peptides with trivalent cations reduces the net charge of the molecule from −5 to −2. However, for the oversaturated regime (Tb^3+^/LBT ratio higher than 1), the ζ-potential increases until it reaches values close to 0 mV. Therefore, we hypothesize that, after saturation, neutralization of charges takes place, and the neutralization is promoted by the excess free Tb^3+^ cations in solution. Moreover, our hypothesis is supported by the fact that dynamic light measurements suggest that the formation of supramolecular structures is triggered by the presence of uncomplexed excess Tb^3+^ in solution. Repulsive forces between species with negative surface charges (for Tb^3+^/LBT ratios of 1 and lower) lead to a monodisperse population of species in solution. Alternatively, as the uncomplexed concentration of Tb^3+^ increases, free ions can nonspecifically coordinate with negatively charged groups on the side chains of the already associated complexes. Furthermore, this coordination of free ions results in the neutralization of the surface charge of species, which leads to aggregation of individual complexes.

The nanosized structures were imaged by transmission electron microscopy (TEM); their morphologies are shown in Figure 2D. These structures, from a solution containing 100 μM of LBTLLA^5–^ and 400 μM of Tb^3+^ cations, appear as polydisperse fibrillar structures with length from 300 to 400 nm, in agreement with the hydrodynamic diameter observed in the DLS measurements. Peptides can self-assemble in a hierarchical process, where the formation of structures such as α-helices, β-sheets, or β-hairpins precedes the creation of nanostructures, with self-assembly credited to side-chain interactions.^51,56,58,59^ Results obtained here suggest that excess ions and neutralization of charges of species in solution are essential for the formation of well-defined secondary structures. Aggregation of proteins in the presence of trivalent salts observed previously has been attributed to the capacity of cations to neutralize the overall surface charge of molecules, which results in aggregation due to van der Waals and hydrophobic interactions.^60,61^

### Tb^3+^ Distribution in Self-Assembling Supramolecular Structures and Stability of Binding Pocket

ASAXS measurements were taken in order to establish whether Tb^3+^ cations play a direct or indirect role in the formation of supramolecular structures. ASAXS allows the concentration of Tb^3+^ to be determined within the self-assembling structures as well as the ratio between the electron density of these elements and the electron density of the organic structures. ASAXS analysis requires a larger signal-to-noise ratio than other scattering characterization techniques such as conventional SAXS, small-angle neutron scattering (SANS), and DLS. To achieve this, the concentration of peptide and Tb^3+^ used for this analysis was eight times greater than the concentrations described above. Solutions containing 800 μM of peptide, and Tb^3+^ cations with concentrations of 3.2, 4.8, 6.4, and 8 mM were prepared by adding TbCl_3_ into peptide solutions. While to the naked eye solutions containing only peptide appear to be clear (Figure 3A), when pipetting a concentrated solution of TbCl_3_, the instantaneous formation of microstructures can be observed (see Figure 3B). These aggregates can sediment over time, as shown in Figure 3C for a solution containing 800 μM peptide and 3.2 mM Tb^3+^ cations, which represent a Tb/peptide ratio of 4. Figure 3D shows the nanometer-sized structures resulting from the Tb^3+^-induced condensation phenomenon, imaged by using TEM. The image shows the presence of amorphous granular aggregates. These amorphous structures might be the result of nucleation of individual fibrils clumping together, similar to what has been observed for amyloid fibrils nucleation.^62–65^

**Figure 3:**
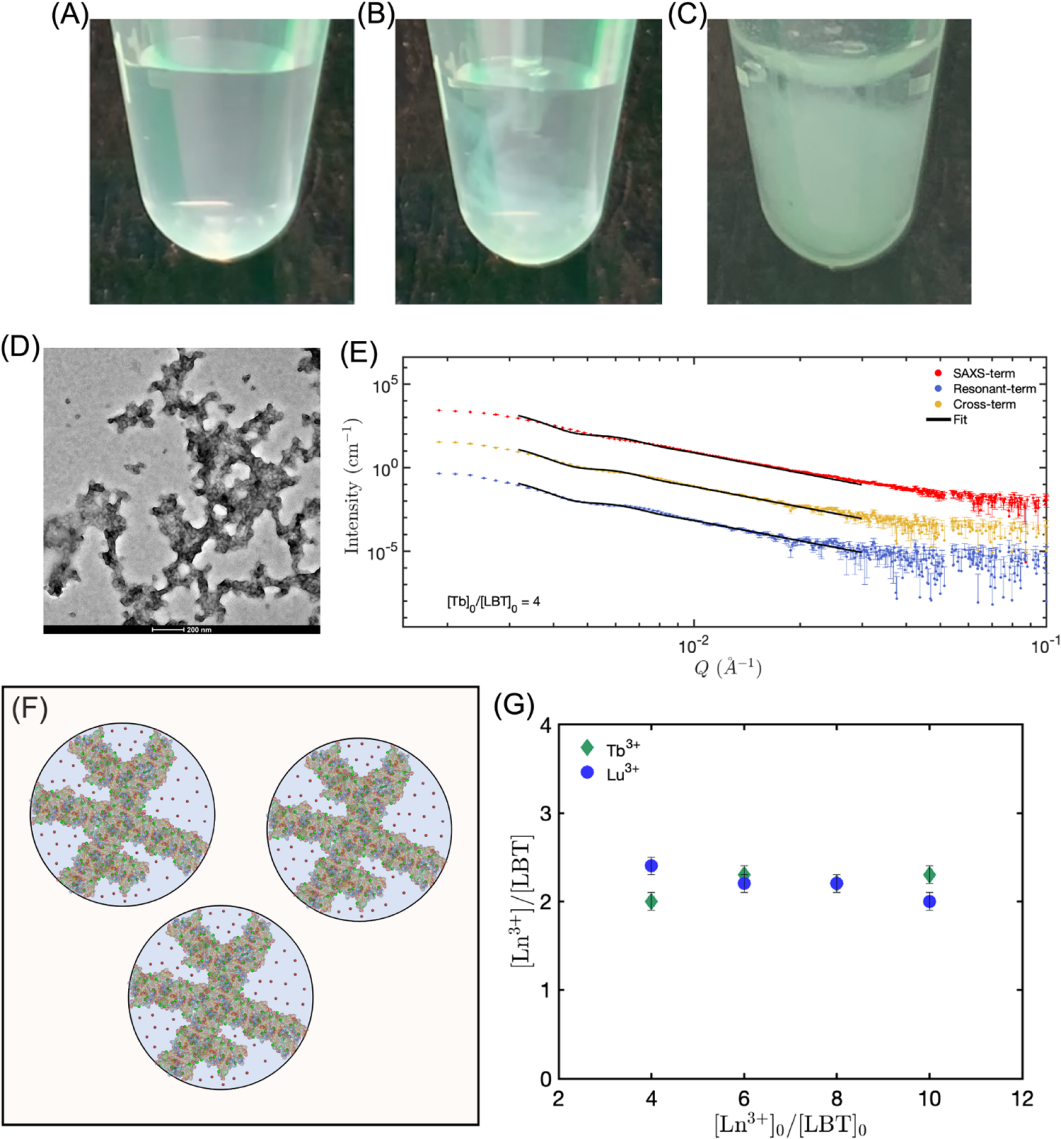
(A) Solution containing 800 μM of LBTLLA^5–^. (B) Aggregation formation when a concentrated solution of Tb^3+^ is added to a solution containing 800 μM of LBTLLA^5–^. (C) Sedimentation of aggregates in a solution containing 800 μM LBTLLA^5–^ and 3.2 mM of Tb^3+^. (D) TEM image from solution containing 800 μM LBTLLA^5–^ and 3.2 mM Tb^3+^; similar structures were observed for Tb^3+^ concentrations higher than 3.2 mM. (E) Representative ASAXS profiles and the corresponding fits for a biphasic spherical model from a solution containing 800 μM LBTLLA^5–^ and 3.2 mM of Tb^3+^. (F) Schematic representation of the biphasic spherical model used for fitting the ASAXS data. The model consists of a peptide–cation phase (a network of Tb-bound peptides, with ions shown in green) and a cation-rich phase (Tb cations shown in red). A third phase permeates the entire spherical structure, comprising bulk water, water molecules bound to peptide-metal complexes, and water molecules associated with free ions. (G) Number of Tb^3+^ and Lu^3+^ cations per LBTLLA^5–^peptide within supramolecular structures as a function of the ratio of added Ln^3+^/LBTLLA^5–^ for a fixed concentration of peptide of 800 μM. Error bars in panel (G) were determined by mapping the chi-squared space.

ASAXS analysis provides insight into the distribution of trivalent cations within these condensed structures. Figure 3E displays ASAXS profiles of amorphous aggregates in solution, along with corresponding fitting curves, for a solution containing 800 μM peptide and 3.2 mM Tb^3+^ cations. The terms presented in Figure 3E are derived from the total scattering intensity *I*(*Q,E*), where *Q* denotes the scattering vector and *E* represents the incident photon energy (see Figure S2A).

The ASAXS scattering intensity as a function of *Q* and *E* forms a system of linear equations (eq S4), which, when solved, determines the scattering components. This system must satisfy the Cauchy-Schwarz inequality (eq S8), setting a lower bound for the resonant term when SAXS and cross-terms are known. At a fixed *Q*, the measured intensity exhibits a linear relationship with the scattering factor *f*′(*E*), increasing as *f*′(*E*) increases (Figure S2B, for a solution containing 800 μM peptide and 3.2 mM Tb^3+^ cations). This linearity reflects the consistent dependence of the intensity on scattering factors, enabling straightforward isolation of the scattering terms. It arises from the contributions of the cross-term and resonant term, both of which are linked to *f*′(*E*). Specifically, the cross-term is proportional to *f*′(*E*), while the resonant term scales with *f*′(*E*)^2^. Over a narrow energy range where the resonant contribution remains relatively small, the intensity follows a predictable linear increase with *f*′(*E*), reinforcing the well-defined relationship between intensity and scattering factors. A detailed explanation of the ASAXS data reduction process is provided in the Supporting Information. Additional ASAXS profiles for varying Tb^3+^ concentrations are shown in Figure S3.

The granular morphology of the aggregates observed in Figure 3D motivated the use of a homogeneous spherical model to extract structural and compositional information from the ASAXS data. This analysis revealed an unreasonably high lanthanide-to-peptide molar ratio, exceeding 20, indicating that the molar concentration of cations within the scattering structures is significantly greater than that of LBT peptides, an unlikely scenario for the short LBTLLA peptide. To better represent the observed system, a “spherical biphasic model” was employed, wherein the aggregates form an extended network with Tb^3+^ cations distributed both within and outside the self-assembled structures (see Figure 3F). In this model, the spherical structures consist of two distinct phases: (1) a “peptide-cation phase” containing LBTLLA:Ln complexes and (2) a “cation-rich phase” composed solely of lanthanide ions. Additionally, a third phase, bulk water, permeates the entire structure but is explicitly accounted for in the model, with its volume fraction defined as 1 minus the sum of the volume fractions of phases 1 and 2.

ASAXS data reduction enables quantification of the Tb^3+^ concentration in both the peptide–cation phase and the cation-rich phase. Moreover, the fitted concentration of the peptide structures allows for the determination of the total number of Tb^3+^ ions per LBT molecule. The electron density profiles for all LBTLLA^5–^:Tb^3+^ solutions are provided in Figure S7A, while the Tb^3+^ concentration profiles are shown in Figure S8A. Fitting parameters are summarized in Table S1. Errors in the fitted parameters are obtained by mapping the chi-squared space (square deviation between the scattering measurement and fit for a given parameter set), which allows for an assignment of the errors in the calculated parameters.

Structural studies indicate that each LBT peptide molecule can coordinate with one lanthanide cation (as represented in Figure 1B), which results in the formation of a pocket that wraps the ion with acidic groups and exposes hydrophobic faces of the molecule.^39,66^ However, a number between 2 and 2.5 Tb^3+^ cations per peptide was obtained for the different concentrations of trivalent cations studied at a constant peptide concentration of 800 μM (see Figure 3G). This ion-to-peptide ratio suggests that at a high concentration of peptide, additional Tb^3+^ cations electrostatically bind with already associated LBTLLA^5–^:Tb^3+^ complexes. Spatial distribution of Lu^3+^ cations was also determined by ASAXS for solutions containing 800 μM peptide and Lu^3+^ cations with concentrations of 3.2, 4.8, 6.4, and 8 mM. ASAXS profiles of these solutions are provided in Figure S4, with electron density profiles given in Figure S7B, Lu^3+^ concentration profiles given in Figure S8B, and fitting parameters given in Table S2. For the different concentrations of trivalent cations studied, a number between 2 and 2.5 Lu^3+^ cations per peptide was obtained, as shown in Figure 3G. Thus, free Lu^3+^ in solution can induce a secondary binding, with LBTLLA^5–^:Lu^3+^ complexes causing aggregation of cation-peptide complexes similar to the Tb^3+^ assemblies.

### Molecular Dynamics Simulations of LBTLLA^5–^:Tb^3+^ Aggregation

Figure 4 shows the conformations of self-assembled structures composed of LBTLLA^5–^:Tb^3+^ complexes in solution derived from all-atom MD simulations. These simulations were initiated with LBTLLA^5–^:Tb^3+^ conformations obtained after microsecond simulations (Figure 1 ^57^). They were conducted both without excess ions (containing 5 LBTLLA^5–^:Tb^3+^ complexes at a 1:1 ratio of cation to peptide; Figure 4A) and with excess ions (including 5 LBTLLA^5–^:Tb^3+^ complexes and 3 free Tb^3+^ ions; Figure 4B) at concentrations comparable to experimental conditions. The self-assembled structures were allowed to equilibrate for approximately a microsecond when steady coordination was observed.

**Figure 4:**
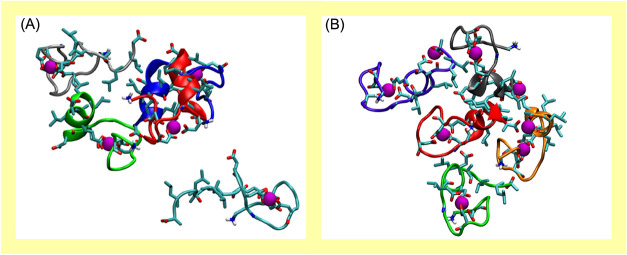
MD simulations snapshots of self-assembling structures from a system containing single LBTLLA^5–^:Tb^3+^ complexes with (A) no free Tb^3+^ in solution and (B) excess Tb^3+^ in solution. Tb^3+^ ions are depicted in magenta, while the peptide backbones are shown in various colors for each molecule to enhance visualization.

In both systems (with and without excess Tb^3+^), aggregation is promoted by hydrophobic interactions between the side chains of the peptide, primarily by noncovalent intermolecular interactions between the amino acids in the hydrophobic block (LLALLA) of the peptide. It is important to note that while the initial hydrophobic segment (LLA) enhances the peptide’s binding affinity for Tb^3+^, as demonstrated by Imperiali et al.,^37^ the addition of a second LLA segment (forming LLALLA) reduces overall metal-binding affinity, though the trend in relative affinity across the lanthanide series remains intact.^57^ Simulations with excess Tb^3+^ in solution indicate that these free cations electrostatically bind to negatively charged, already associated LBTLLA^5–^:Tb^3+^ complexes. Two types of binding were observed: one negatively charged complex can bind with an additional free ion or two bound peptides can electrostatically bind with an additional free ion, forming a bridge between the two complexes. Simulations suggest that this electrostatic binding occurs through negatively charged groups that are not involved in the selective binding loop (specifically D11 and the COO^–^ group from the C-terminus). The secondary complexation results in aggregation through the bridging of complexes in solution, enhancing the self-assembly of individual complexes, as shown in Figure 4B. Simulations indicate a lower degree of aggregation in the absence of excess ions (Figure 4A), which aligns with the visual observation of condensates at higher concentrations of free lanthanides in solution.

The final conformations of the aggregated complexes reveal that LBTLLA^5–^ can aggregate into nanostructures rich in trivalent cations, with excess Ln^3+^ driving self-assembly through electrostatic bridging. Notably, this secondary association occurs only with carboxylate groups from the aspartic acid (D11) and the C-terminus, which do not participate in the selective binding loop. Consistently, MD simulations indicate that all LBTLLA^5–^:Tb^3+^ complexes within the aggregated structures, whether involved in electrostatic bridging or not, retain their compact formations, wrapping the Tb^3+^ ion as in the monomeric state.

### Selective Binding of Ln^3+^ Cations with LBT Peptides in Supramolecular Structures

We have demonstrated that LBTLLA^5–^ associates with lanthanides and retains these ions in their binding pockets upon aggregation, even in the presence of excess free metals, which induce a secondary binding. To understand how this secondary binding influences selectivity and separation through macromolecular assembly, we now investigate the LBTLLA^5–^ complexation from mixed aqueous feedstocks to identify factors that could potentially influence separation. We begin with solutions containing 800 μM peptide and equimolar mixtures of two lanthanides, terbium and lutetium. These metals were chosen for the competitive study to focus on the separation of two heavy lanthanides, a particularly challenging task given their closely related chemical characteristics. Importantly, the peptide exhibits one of the largest differences in dissociation constants between these two elements within the heavy lanthanide series.^57^ This distinct disparity makes Lu^3+^ an ideal counterpart to Tb^3+^ for revealing clear and measurable differences in metal-peptide complexation, aggregation, and interfacial behavior. The total lanthanide concentration in these equimolar mixtures varies from 3.2 to 8 mM, ensuring all LBTLLA^5–^ is complexed. These concentrated conditions, defined as the upper “saturation” regimes, are necessary for aggregation and subsequent condensation as explained below.

The distribution of trivalent cations in a mixture containing equimolar concentrations of Tb^3+^ and Lu^3+^, with a constant concentration of peptide, was determined by ASAXS. Scattering profiles of these solutions are shown in Figures S5 and S6, with electron density profiles in Figure S7C,D, Lu^3+^ concentration profiles in Figure S8C,D, and fitting parameters in Tables S3 and S4. The number of Tb^3+^, Lu^3+^, and total Ln^3+^ cations per peptide are represented in Figure 5A. These ratios were also confirmed by using ICP-OES and UV absorption spectroscopy (see Figure 5B), validating the results obtained from fitting the X-ray scattering data from the ASAXS technique.

**Figure 5:**
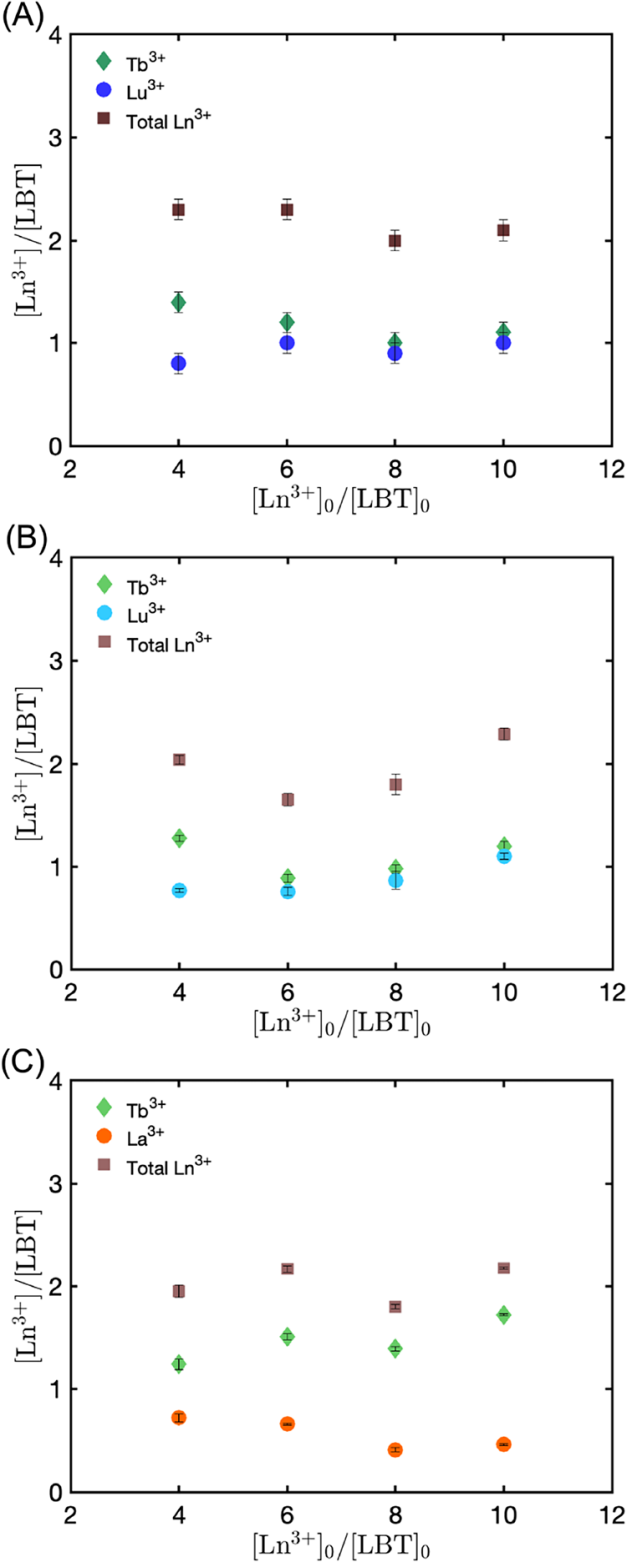
Number of individual and total Ln^3+^ cations per LBTLLA^5–^ peptide within supramolecular structures as a function of the ratio of added Ln^3+^/LBT for solutions containing binary equimolar concentrations of Ln^3+^ and a fixed peptide concentration of 800 μM for (A) Tb^3+^ and Lu^3+^ determined by ASAXS, (B) Tb^3+^ and Lu^3+^ determined by ICP-OES, and (C) Tb^3+^ and La^3+^ determined by ICP-OES. Error bars of individual ions in panel (A) are determined by mapping the chi-squared space. Error bars for individual ions in (A) were obtained by mapping the chi-squared space; those for total Ln^3+^ in (A) were calculated by propagating the uncertainties from this mapping. Error bars in (B, C) reflect error propagation from the standard deviations of three independent absorbance and ICP-OES measurements.

Size distribution and peptide concentration were assessed after redispersion of the peptide-metal pellet in buffer to a final volume of 10 mL (8 μM peptide). Figure S9, corresponding to the redispersed pellet from a solution containing 800 μM peptide and 3.2 mM Tb^3+^, shows that the metal-peptide complexes predominantly exist in the monomeric state. UV–vis absorbance at 280 nm measured 0.065 ± 0.002, indicating that the peptide concentration after redispersion remained within 2% of the initial value prior to aggregation and centrifugation. These results demonstrate that the aggregates are fully reversible upon dilution and mechanical disruption, and that the aggregation process is driven by noncovalent, dynamic interactions, primarily hydrophobic and electrostatic, rather than by irreversible structural changes.

The total number of cations per peptide for the mixture of Tb^3+^ and Lu^3+^ is consistent with the results obtained from structures formed with only one type of ion. This indicates that secondary association also occurs when two ions coordinate simultaneously with the peptide. Importantly, this secondary association is independent of ion size, hydration state, or acidity, and thus lacks selectivity across the lanthanide series compared to primary coordination that forms the binding loop. Furthermore, based on the experimental Ln^3+^/LBTLLA^5–^ ratios, complexed ions that do not form part of the selective binding loop account for up to approximately 62% of the total Ln^3+^ density within the self-assembling structures. These findings suggest that the selectivity of the peptide may be affected by the secondary association, which is driven by interactions of Ln^3+^-O with the aspartic acid (D11) residue and the C-terminus of the biomolecule.

Separation factors from ASAXS, the ratio of Tb^3+^ to Lu^3+^, are provided in Table 1. Ratios were also calculated by isolating the aggregates from the solution through centrifugation and measuring the metal concentrations using inductively coupled plasma optical emission spectroscopy (ICP-OES) along with the concentration of LBTLLA^5–^ using ultraviolet–visible (UV–vis) absorption spectroscopy. These values, which are also reported in Table 1, are all in good agreement. For the lowest total Ln^3+^ concentration in solution, the Tb^3+^/Lu^3+^ ratio within the structures is consistent with the expected value of approximately 1.6, based on association constants.^57^ This agreement suggests that the secondary coordination observed in the aggregated state may not significantly impact the peptide’s affinity for the lanthanide series compared to the monomeric, diluted state. However, as the total Ln^3+^ concentration increases, the Tb^3+^/Lu^3+^ ratio decreases, contrary to what the association constants indicate. This greater concentration of Lu^3+^ could be attributed to a greater number of free Lu^3+^ ions that do not form part of the stable selective binding pocket because of the lower affinity of this metal with the peptide. The higher concentration of Lu^3+^ might also result from secondary binding that favors Lu^3+^ over Tb^3+^ due to Lu^3+^’s greater Lewis acidity, which makes it more effective at accepting electron pairs from donor atoms in the ligand. Since the results for the Tb^3+^-Lu^3+^ pair are not entirely conclusive, mixtures of cations with more distinct association constants might provide a better understanding of the observed differences in selectivity.

**Table 1:** Tb^3+^/Lu^3+^ and Tb^3+^/La^3+^ Ratios as a Function of the Ratio of Added Ln^3+^/LBTLLA^5–^ Determined from ASAXS and ICP-OES for a Fixed Peptide Concentration of 800 *μ*M^a^

[Ln^3+^]_0_/[LBT]_0_	Tb^3+^/Lu^3+^ ASAXS	Tb^3+^/Lu^3+^ ICP-OES	Tb^3+^/La^3+^ ICP-OES
4	1.7 ± 0.2	1.67 ± 0.06	1.7 ± 0.1
6	1.2 ± 0.1	1.17 ± 0.08	2.27 ± 0.05
8	1.1 ± 0.1	1.1 ± 0.1	3.4 ± 0.1
10	1.1 ± 0.1	1.09 ± 0.06	3.72 ± 0.06

a Error bars of ratios calculated from ASAXS were calculated by propagating the uncertainties from mapping the chi-squared space. Error bars of ratios calculated from ICP-OES reflect error propagation from the standard deviations of three independent absorbance measurements and ICP-OES measurements.

Despite the efficacy of ASAXS measurements in determining cation distribution within peptide self-assembly structures in situ, the technique cannot be applied at high lanthanide concentrations using energies below 7 keV. This limitation arises because the experimental facility’s lowest available energy for ASAXS measurements is 5.5 keV. Additionally, samples with high concentrations of lanthanides excessively absorb X-rays, especially near the absorption edges, resulting in inadequate signal-to-noise ratio data. A high signal-to-noise ratio is essential for reliable ASAXS measurements. Light lanthanides with low X-ray absorption energies, such as those with association constants lower than Tb^3+^ and Lu^3+^, cannot be measured because their absorption edges are lower than 7 keV, and increased X-ray absorption at lower energies further compromises scattering data quality. In general, X-ray scattering from ions with ionic radii larger than Sm^3+^ cannot be assessed with ASAXS due to the unavailability of lower energies and excessive X-ray absorption at low energies. On the other hand, we have demonstrated that ICP-OES and UV absorption spectroscopy are effective for ex situ measurements of the concentration of peptide and lanthanide ions within self-assembling structures. Therefore, we investigated the selective binding between terbium and lanthanum using solutions containing 800 μM of peptide and equimolar mixtures of the two metals in upper saturation regimes (3.2 mM to 8 mM, similar to the conditions used for Tb^3+^ and Lu^3+^). Lanthanum was selected because it has the lowest affinity for LBTLLA^5–^ among the lanthanides, while Tb^3+^ has the highest affinity.^57^ This competitive separation study between Tb^3+^ and La^3+^ is expected to provide deeper insights into the selective binding of metals under aggregated conditions.

Figure 5C shows the number of Tb^3+^, La^3+^, and total Ln^3+^ ions per peptide as a function of the total cation concentration in solution. These results are consistent with those obtained for the aggregated structures coordinating with individual ions as well as with mixtures of Tb^3+^ and Lu^3+^, supporting the presence of secondary association, as indicated by the number of ions per peptide in the nanostructures.

The Tb^3+^/La^3+^ ratios as a function of the initial total lanthanide to peptide concentration in solution ([Ln^3+^]_0_/[LBT]_0_) are presented in Table 1. For the lowest total Ln^3+^ concentration, the Tb^3+^/La^3+^ ratio within the aggregates closely resembles the ratio obtained for Tb^3+^/Lu^3+^, which is unexpected based on the association constant measured in the diluted regime (Tb^3+^/La^3+^ is approximately 35).^57^ Additionally, as [Ln^3+^]_0_/[LBT]_0_ increases, a higher concentration of Tb^3+^ is observed. This contradicts the notion that monomeric selectivity diminishes as the concentration of the ion with the lower affinity constant increases. While the concentration of the metal with greater Lewis acidity (Tb^3+^) increases with the total lanthanide concentration, consistent with the higher concentration of Lu^3+^ in the terbium–lutetium mixture, the resulting Tb^3+^/La^3+^ ratios suggest an excess concentration of La^3+^ within the aggregates containing both terbium and lanthanum.

The unexpected cationic densities could be associated with the intermolecular interactions between individual LBTLLA^5–^:Tb^3+^ complexes, which result in aggregate formation through electrostatic and hydrophobic interactions absent in the diluted monomeric regime. However, visual inspection of single-component solutions revealed that the density of visible aggregates depends not only on the total concentration of added lanthanides (with greater condensates forming at higher metal concentrations) but also on the specific ion added. This suggests a degree of metal dependency in the aggregation of complexes, affecting the metal density within the self-assembly structures and, thus, the selective separation. Note that this metal-dependent aggregation can also affect the ASAXS measurements since the scattering intensity is generally more sensitive to larger structures in solution due to their greater mass and volume, which contribute more significant to the overall scattering signal. As a result, the ASAXS data may predominantly reflect the metal content within larger aggregates.

To provide direct experimental evidence of metal dependency in aggregation and separation, we quantified the peptide concentration (measured by UV–vis absorption spectroscopy) of isolated aggregates in single-component systems from solutions containing 800 μM of LBTLLA^5–^ and Tb^3+^, Lu^3+^, or La^3+^ in upper saturated regimes (3.2 mM to 8 mM). These values are reported in Figure 6 as the extracted percentage of LBTLLA^5–^. The results demonstrated the expected metal dependency in separation, indicating that the extracted percentages for LBTLLA^5–^:La^3+^ and LBTLLA^5–^:Lu^3+^ are greater than for LBTLLA^5–^:Tb^3+^ (with the order of separation being LBTLLA^5–^:La^3+^ > LBTLLA^5–^:Lu^3+^ > LBTLLA^5–^:Tb^3+^). This metal dependence explains the higher density observed for Lu^3+^ and La^3+^ compared to the more selective Tb^3+^ in two-component mixtures.

**Figure 6:**
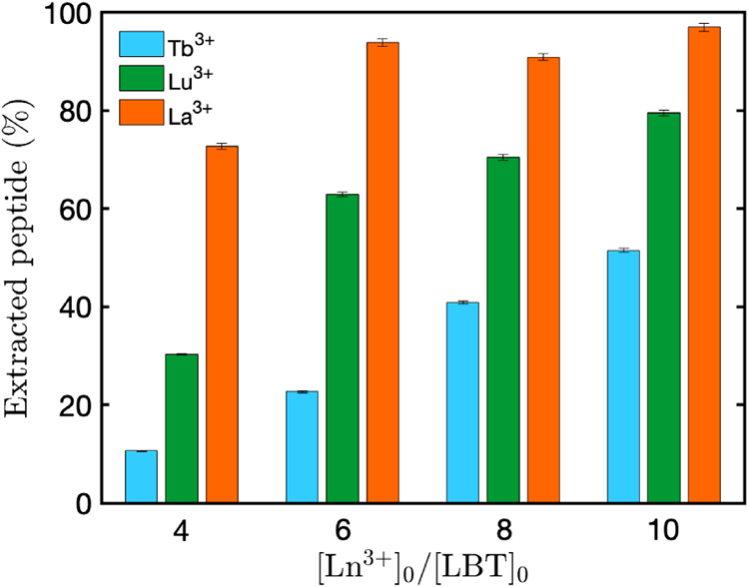
Percentage of extracted LBTLLA^5–^ peptide from separated aggregates as a function of total [Ln^3+^]_0_/[LBT]_0_ ratios for single-component solutions containing 800 μM peptide and only one type of cation (Tb^3+^, Lu^3+^, or La^3+^) for different concentrations of lanthanides. The extracted peptide percentage is determined as the ratio of peptide concentration in the pellet to the total initial peptide concentration solution, calculated from UV–vis measurements. Error bars were calculated by propagating the standard deviations of three independent absorbance and ICP-OES measurements.

It is important to note that while the metal-triggered condensation of these structures is performed in a batch-like manner, where centrifuged pellets are separated from the supernatant for redispersion and stripping, the process can also be adapted to continuous flow using decanter centrifuges. In this setup, the denser peptide-lanthanide aggregates would be continuously separated and collected, while the lighter supernatant is discharged, enabling uninterrupted operation. This approach has been successfully demonstrated in the context of protein aggregate separation; for example, continuous centrifugation has been used to efficiently separate whey protein aggregates.^67,68^

To demonstrate the capability of the peptide to rebind lanthanide ions after acid-induced stripping, we performed a series of binding-desorption cycles under controlled solution conditions. While this study does not include the physical separation or centrifugation steps, it provides fundamental insights into the reversibility and stability of the peptide-metal interaction, key properties for any reusable extraction system.

We monitored the peptide’s ability to rebind terbium across multiple cycles using fluorescence spectroscopy, with tryptophan excitation at 280 nm and terbium emission measured at 545 nm. A solution containing 100 μM peptide and 400 μM Tb^3+^ was subjected to five successive binding-stripping cycles. In each cycle, Tb^3+^ was desorbed by lowering the pH to 2 using HCl, followed by readjusting to pH 5.8 with 1 M NaOH to allow rebinding. The fluorescence signal remained relatively stable across the first four cycles, and by the fifth cycle, approximately 92% of the initial binding signal was retained (Figure S10). These results indicate that the peptide can undergo multiple binding-release events with minimal loss of function, highlighting its chemical robustness and potential suitability for future development into regenerative lanthanide capture systems.

It is important to consider that in feedstock mixtures monovalent and divalent metal ions such as Na^+^, Mg^2+^, and Ca^2+^ are typically present at much higher concentrations than lanthanides and could potentially interfere with selective separation processes. We could not evaluate their separation via precipitation due to the absence of peptide precipitation in their presence, even at high concentrations. However, this lack of precipitation is advantageous, as it suggests that even if these ions exhibit some degree of binding to the peptide, they do not contribute to the formation of insoluble peptide–metal macrostructures and would therefore remain in solution during sedimentation of the peptide–lanthanide complexes. To further assess the selectivity, we investigated the potential competitive binding of Na^+^ and Mg^2+^ using fluorescence spectroscopy. Terbium binding was monitored via its characteristic emission at 545 nm, sensitized by tryptophan excitation at 280 nm. In peptide–Tb^3+^ mixtures (100 μM peptide, 400 μM Tb^3+^), the addition of up to 1 M Na^+^ or Mg^2+^ caused no measurable decrease in emission intensity (Figure S11A,B), indicating that these cations do not displace Tb^3+^ from the peptide at high concentrations. Together, these results highlight both the strong binding preference of the peptide for lanthanides and the benefit of nonco-precipitating competing ions, supporting the peptide’s potential as a highly selective bioextractant in complex ionic environments.

## Conclusions

This work has explored the use of the lanthanide-binding tag peptide LBTLLA^5–^ as an extractant for REEs from aqueous feedstocks containing mixtures of Ln^3+^. We demonstrated that lanthanide cations can induce aggregation and subsequent condensation of negatively charged peptide LBTLLA^5–^ at ambient temperature. This spontaneous condensation can be exploited for an all-aqueous, eco-friendly separation process, facilitated by the ease of separating REE-rich condensates from the feedstock solution via centrifugation. We found that the selective separation of REEs using the LBTLLA^5–^ peptide in a macromolecular assembly platform depends not only on the selective coordination of the resulting LBT:Ln^3+^, which forms a stable binding loop, and on the excess negative charge of the LBT:Ln^3+^, which can nonspecifically associate with excess ions in the feedstock solutions, but also on the degree of aggregation of individual LBT:Ln^3+^ complexes. This aggregation can be controlled by (1) the concentration of excess lanthanides in solution, with greater aggregation occurring at higher metal concentrations and (2) the type of lanthanide with which the peptide complexes, ranked in aggregation propensity as La^3+^ > Lu^3+^ > Tb^3+^. LBT molecules are excellent candidates for the separation of these metals because they can be optimized to selectively coordinate with high affinity for particular Ln^3+^ ions. We demonstrated that this separation can also be tuned by manipulating the lanthanide-to-peptide ratio in solution. Moreover, nonselective interactions resulting from the excess charge of the complexed molecule can be engineered by modifying these groups to create partial binding pockets that, together with the partial binding pocket of another LBT molecule, can selectively coordinate with one lanthanide ion and promote self-assembly. The results presented in this work lay the foundation for further engineering of LBT peptides to improve the selectivity and separation of REEs by tuning electrostatic bridging, charge neutralization, and hydrophobic interactions. Furthermore, the ability of the Ln^3+^-peptide assemblies to form networks resulting in the condensation of micrometer-sized structures enables the use of low-energy separation methods such as sedimentation and microfiltration to isolate the desired elements from impurities or undesired elements present in feedstock solutions.

## Materials and Methods

### Materials

LBTLLA^5–^: YIDTNNDGWYEGDELLALLA (purity ≥ 95%) labeled at the N-terminus with a free amine and labeled at the C-terminus with a free acid was purchased from GenScript (Piscataway, NJ, USA), diluted to a stock concentration of 1 mM in buffer solutions containing 100 mM NaCl (purity ≥ 99.5%, Sigma-Aldrich) and 50 mM MES (purity ≥99.5%, Sigma-Aldrich) at a pH of 6, and used without additional purification. TbCl_3_ hexahydrate (purity ≥99.999%), LuCl_3_ hexahydrate (purity ≥99.99%), and LaCl_3_ heptahydrate (purity ≥99.99%) were purchased from Sigma-Aldrich and diluted to a stock concentration of 25 mM in the same buffer solution as for the peptide containing 100 mM NaCl and 50 mM MES. Buffer solution is filtered using a 0.22 μm poly­(tetrafluoroethylene) filter. Ultrapure water is obtained from a Milli-Q water filtration unit (EMD Millipore) with a resistivity of 18.2 MΩ and used for the preparation of buffer solution.

### Dynamic Light Scattering

DLS was performed on a Zetasizer Nano ZS instrument (Malvern). 1 mL of the different solutions containing 100 μM LBTLLA^5–^ and different concentrations of Tb^3+^ ranging from 0 mM to 8 mM were analyzed in plastic cuvettes at 25 °C. The z-average diameter and polydispersity index (PDI) were calculated from a cumulants analysis, where the diffusion coefficient of particles is converted into a particle size by using the Stokes–Einstein equation.

### ζ-Potential Measurements

ζ-Potential measurements were taken by using a Zetasizer Nano ZS (Malvern). 700 μL of the different solutions containing 100 μM LBTLLA^5–^ and different concentrations of Tb^3+^ ranging from 0 mM to 8 mM were loaded in folded capillary cells and analyzed at a temperature of 25 °C. Electrophoresis measurements were calculated based on the movement of the particles under the influence of an applied electric field relative to the liquid where they are suspended. ζ-Potential measurements were then computed by using Henry’s equation and the electrophoretic mobility values, under conditions where the Debye length is small compared to the particle radius (Smoluchowski limit, *F*(*ka*) = 1.5).

### Transmission Electron Microscopy

TEM measurements were undertaken on a Tecnai Spirit TWIN TEM electron microscope operated at an accelerating voltage of 120 kV. Solutions were prepared with different concentrations of LBTLLA^5–^ and Tb^3+^ cations, and 4 μL samples were deposited onto TEM grids (pure carbon on copper mesh, Ted Pella, Inc., USA) that were previously treated with a plasma cleaner (Fischione M1070 NanoClean) for 60 s. The sample on the grid was lightly blotted with filter paper and then stained with 2% uranyl acetate solution and blotted once again. The sample was rinsed with water, and the excess solution was removed by blotting the edge of the grid with filter paper.

### Anomalous Small-Angle X-ray Scattering

The ASAXS measurements were taken at the NSF’s ChemMatCARS (15-ID-D) beamline of Advanced Photon Source at Argonne National Laboratory. Solutions containing 800 μM of LBTLLA^5–^, and different concentrations of TbCl_3_ and LuCl_3_ at pH 6, with 50 mM MES buffer and 100 mM of NaCl were loaded in 0.05 in. diameter polyimide tubes. Data frames were collected with 1 s exposure time using a Pilatus3 × 300 K detector with a 1 mm Si chip and a sample-to-detector distance of 3.6 m. ASAXS data was collected at 20 different energies below the X-ray absorption L_3_ edge of Tb and Lu (7.514 and 9.244 keV, respectively). The scattering patterns were also collected from a solution containing just the trivalent salt with similar concentrations as the samples for background subtraction and glassy carbon for absolute scale normalization at the same energies as the sample. Different scattering terms (*SAXS-term*, *Cross-term*, and *Resonant-term*) were obtained from energy-dependent SAXS data following the same process described elsewhere^69,70^ and in the section “ASAXS data reduction using Stuhrmann method” presented in the Supporting Information. To identify the distribution of counterions, a biphasic model named as “Biphasic Sphere Uniform” function was developed within XModFit, a data modeling software developed by NSF’s ChemMatCARS (https://github.com/chemmatcars/XModFit.git)^71^ and used to fit all the scattering terms simultaneously for obtaining various metrical information.

Biphasic Sphere Uniform function: This function calculates different scattering contributions (SAXS-term, Cross-term, and Resonant-term) from a spherically symmetric structure composed of two different phases of solute with specified volume fractions in a solvent. Combining the information from TEM, we have approximated the aggregates to form spherical structures (Figure 3F in the main article), which are composed of three distinct phases: (1) peptide-Ln^3+^ complex-rich phase with volume fraction *Phase1*_*volFrac*, (2) LnCl_3_-rich aqueous phase with volume fraction *Phase*2_*volFrac*, and (3) the aqueous phase with volume fraction 1 – *Phase*1_*volFrac* – *Phase*2_*volFrac*. To include the polydispersity, the scattering patterns are averaged over a log-normal distribution of radii of the spherical aggregates. Fitting with this model was performed by varying the density of self-assembling structures (norm), the density (*Phase*1_*Density*) and volume fraction (*Phase*1_*volFrac*) of the peptide-Ln^3+^ phase (*C*103*H*149*N*23*O*36*LnPhase1*_*rmoles*) with *Phase*1_*rmoles* being the number of lanthanide ions per peptide, the density (*Phase*2_*Density*) and volume fraction (*Phase*2_*volFrac*) of the LnCl_3_ rich phase, and the mean radii (*Phase*1_*R*) and width of the radii (*Rsig*) distribution of the spherical self-assembled structures. The fits of all of the scattering components obtained from ASAXS data are shown in Figures S2–S5. All of the parameters obtained after fitting are tabulated in Tables S1–S4.

### Inductively Coupled Plasma Optical Emission Spectroscopy

ICP-OES measurements were taken by using a Shimadzu ICPE-9000 spectrometer. Solutions containing 800 μM LBTLLA^5–^ and different concentrations of Tb^3+^ and Lu^3+^ or Tb^3+^ and La^3+^ were centrifuged at 12,000 r.p.m. for 30 min after 2 h of preparation. The supernatant was discarded, and the precipitate was washed with a buffer solution, followed by redispersion of the solid in the same buffer solution. Calibration solutions were prepared from certified stocks of each metal (SCP Science, Montreal, Canada) prior to the measurements. The instrument was calibrated using a five-point calibration curve between 0.05 and 1 mg/L and checked by three QC samples at the low, middle, and high points on the curve. The operating conditions employed for ICP-OES determination were as follows: 1200 W of RF power, 10 L/min of plasma flow, 0.6 L/min of auxiliary flow, 0.7 L/min of nebulizer flow, and 1 mL/min of sample uptake rate.

### Ultraviolet–Visible Absorption Spectroscopy

Absorbance of samples containing LBTLLA^5–^ peptide with or without lanthanide cations was measured using a UV–vis spectrophotometer (Thermo Scientific). Absorbance of the same solutions prepared for ICP-OES analysis was measured at 280 nm. Prior to every measurement, blank calibration of the UV–visible spectrophotometer was done with buffer solution. The concentration of peptide in solution was calculated by using this absorbance value and assuming an extinction coefficient ε = 8250 cm^–1^ M^–1^.^72^

### Luminescence Spectroscopy

Energy transfer between tryptophan (position 7) and Tb^3+^ was monitored using a Jasco FP-8500 spectrophotometer equipped with a 3 mm path length microfluorescence cuvette. Tryptophan was excited at 280 nm, and emission was detected at a wavelength range from 460 to 600 nm for competition assays and a fixed wavelength of 545 nm for cycling studies. Both excitation and emission bandwidths were set to 2.5 nm. Measurements were recorded with a 0.1 s response time, medium sensitivity, a data interval of 1 nm, and a scan speed of 200 nm/min. To evaluate potential displacement of terbium ions by non-rare-earth elements, fluorescence spectra were recorded as a function of Na^+^ and Mg^2+^ concentrations, using a fixed peptide concentration (100 μM) and Tb^3+^ concentration (400 μM). pH cycling experiments were conducted to assess binding reversibility: the pH was first lowered to 2 using 1 M HCl and then readjusted to 5.8 with 1 M NaOH. Fluorescence intensities were corrected for dilution after each cycle.

### Molecular Dynamics Simulations

MD simulations were performed to model the complexed LBTLLA^5–^:Tb^3+^ binding complexes in a concentrated state to study aggregation in aqueous solution using GROMACS package.^73,74^ All peptides were modeled using the CHARMM36 force field.^75^ Terbium cations were modeled using the modified CHARMM force field,^76^ which were designed to match the hydration structure and hydrogen free energy from experimental measurements. The solvent was modeled using the modified Tip3p water model under neutral pH conditions. Unless otherwise stated, we use periodic boundary conditions in the *x*-, *y*-, and *z*-directions. Sodium and chloride ions were used to neutralize the system, and the concentration of NaCl was 100 mM, which is comparable to the experimental conditions. Particle Mesh Ewald algorithm^77^ was adopted for the calculation of long-range electrostatic interactions. The integration time step was set to 2.0 fs, and the LINCS algorithm^78^ was employed to constrain the lengths of all chemical bonds involving hydrogen atoms at their equilibrium values.

The initial configuration of LBTLLA^5–^:Tb^3+^ binding complex was obtained from our previous work^57^ which was acquired by residue mutation using the Scwrl4 program^79^ with starting point being the structure of LBT1:Tb^3+^ binding complex (PDB code: 1TJB^39^). Systems containing 5 LBTLLA^5–^Tb^3+^ complexes and 5 LBTLLA^5–^:Tb^3+^ complexes with 3 free Tb^3+^ ions were allowed to equilibrate for approximately a microsecond until steady coordination was achieved. The solvated system was first energy minimized using the Steepest Descent (SD), while algorithms were used to remove unfavorable contacts. The isochoric isothermal (NVT) simulations were then performed at room temperature of 298 K using a stochastic velocity rescaling algorithm for 5 ns.^80^ After the equilibration stage, isobaric isothermal (NPT) simulations were performed under room temperature and ambient pressure (1 bar), using a velocity rescaling thermostat and a Parrinello–Rahman barostat.^81^

## Supplementary Material



## Notes

Equilibrium concentrations of free peptide and free Tb calculations, intensity distribution from DLS measurements, ASAXS scattering components and fittings, electron density profiles and lanthanides concentration profiles from ASAXS fittings, and ASAXS fitting parameters (PDF)
