# Supplementary material for: Supramolecular Assembly of Lanthanide-Binding Tag Peptides for Aqueous Separation of Rare Earth Elements
Source: ACS Nano. 2025 Oct 6;19(41):36171–83. doi: 10.1021/acsnano.5c05056 (PMC12548334; doi:10.1021/acsnano.5c05056)
Supplement: Supplementary file 1 [file nn5c05056_si_001.pdf]

Supporting Information

Supramolecular Assembly of Lanthanide Binding Tag Peptides for Aqueous Separation of Rare Earth Elements

Luis E. Ortuno Macias[1,2], Felipe Jiménez-Ángeles[3], Surabh S. KT[1], Kathleen J. Stebe[4], Monica Olvera de la Cruz[3], Mrinal K. Bera[5], Wei Bu[5], Binhua Lin[5], Charles Maldarelli[*,1,2], and Raymond S. Tu[*,1]

[1]Department of Chemical Engineering, The City College of New York, New York, 10031, NY, USA
[2]Levich Institute, The City College of New York, New York, 10031, NY, USA
[3]Department of Materials Science and Engineering, Northwestern University, Evanston, 60208, IL, USA
[4]Department of Chemical and Biomolecular Engineering, University of Pennsylvania, Philadelphia, 19104, Pennsylvania, USA
[5]NSF's ChemMatCARS, Pritzker School of Molecular Engineering, University of Chicago, Chicago, 60637, IL, USA

[*]Corresponding authors: cmaldarelli@ccny.cuny.edu; tu@ccny.cuny.edu

Equilibrium concentrations of free peptide and free Tb^{3+}

The equilibrium concentrations of free $LBTLLA^{5-}$ and free Tb^{3+} were determined using the dissociation constant (K_D) of the reaction LBTLLA + Tb ↔ LBTLLA:Tb.[1] Given an initial peptide concentration [$LBTLLA_0$] = 100 μM and a range of initial Tb^{3+} concentrations [Tb_0] from 0 to 1000 μM, the equilibrium concentrations were calculated by solving the mass balance equations from LBTLLA and Tb. The total peptide balance is given by [LBTLLA] = [$LBTLLA_0$] - [LBTLLA:Tb], and the total Tb balance is [Tb] = [Tb_0] - [LBTLLA:Tb]. Substituting the expression [LBTLLA:Tb] = [LBTLLA][Tb]/K_D into the mass balance equations results in a quadratic equation for [LBTLLA] and [Tb], which was solved numerically for each [Tb_0].

ASAXS data reduction using Stuhrmann method

The analysis of ASAXS data was performed using the Stuhrmann method, which allows for the determination of the spatial distribution of specific atomic species (e.g., Ln) within peptide aggregates. This method leverages the variation in scattering contrast as the incident x-ray energy is tuned near the absorption edge of the anomalous element. For centrosymmetric particles, the energy-dependent complex scattering factor, $f(E)$, causes the radial scattering length density profile, ρ_e, of the resonantly scattering units to also become energy-dependent, expressed as[2-4]:

$$\rho_e(\vec{r},E) = \rho_{eo}(\vec{r}) - \rho_{es} + v(\vec{r})(f'(E) + if''(E)) \qquad \text{(S1)}$$

with $\rho_{eo}(\vec{r})$ representing the energy-independent total electron density of the system (e.g., the usual contrast well below the absorption edge of the resonant element), ρ_{es} denoting the electron density of the solvent (water in our case, with a value of 0.334 electrons/Å^3), $v(\vec{r})$ as the number density of the anomalous scattering units (e.g., Ln), and $f'(E)$ and $f''(E)$ as the real and imaginary components of the complex scattering factors of the resonant units, respectively. Taking the Fourier transform of equation S1 yields the x-ray scattering expression defined in equation S2, which can be further simplified into equation S3. Furthermore, for systems with centrosymmetric geometries, the terms become real and isotropic in $\vec{Q}$, allowing the simplification of equation S3 into equation S4.[2-4]

$$I(\vec{Q},E) = \frac{N}{V} r_e^2 \left| \int \rho_e(\vec{r},E) e^{i\vec{Q}.\vec{r}} d\vec{r} \right|^2 \qquad \text{(S2)}$$

$$I(\vec{Q},E) = |\rho_{os}(\vec{r})|^2 + f'(E) Re\left[\rho_{os}(\vec{Q})\rho_r^*(\vec{Q}) + \rho_{os}^*(\vec{Q})\rho_r(\vec{Q})\right] + [f'^2(E) + f''^2(E)]|\rho_r(\vec{r})|^2 \qquad \text{(S3)}$$

$$I(Q,E) = \frac{N}{V}\left[\rho_{os}^2(Q) + 2f'(E)\rho_{os}(Q)\rho_r(Q) + \left(f'^2(E) + f''^2(E)\right)\rho_r^2(Q)\right] \qquad \text{(S4)}$$

with N/V representing the number density of particles per unit volume, $r_e = 2.818 \times 10^{-15}\, m$ the classical electron radius, $\rho_{os}(\vec{r}) = \int (\rho_{eo}(\vec{r}) - \rho_{es}) e^{i\vec{Q}.\vec{r}} d\vec{r}$ and $\rho_r(\vec{Q}) = \int v(\vec{r}) e^{i\vec{Q}.\vec{r}} d\vec{r}$ are complex numbers, Re denotes real part of the complex number. Equation S4 applies to a monodisperse system of particles. For a polydisperse system,

the intensity must be averaged over the particle size distribution, modifying the equation to:

$$I(Q,E) = \frac{N}{V}\left[\langle \rho_{os}^2(Q)\rangle + 2f'(E)\langle \rho_{os}(Q)\rho_r(Q)\rangle + \left(f'^2(E) + f''^2(E)\right)\langle \rho_r^2(Q)\rangle\right] \quad \text{(S5)}$$

$N/V\langle \rho_{os}^2(Q)\rangle = I_S$ is the *SAXS-term*, which is energy independent. $N/V\langle \rho_{os}(Q)\rho_r(Q)\rangle = I_C$ is the *Cross-term*, and $N/V\langle \rho_r^2(Q)\rangle = I_R$ is the *Resonant-term*, contributing only from the resonant element. Both the *Cross-term* and *Resonant-term* are energy-dependent. The values of $f'(E)$ and $f''(E)$ are obtained from the National Institute of Standards and Technology database.[5] The scattering intensity as a function of Q and E from ASAXS measurements forms a system of linear equations based on equation S4, which, when solved, allows to determination of I_S, I_C, and I_R.[4]

In general, the *Resonant-term* in the scattering equation is significantly smaller than the other terms, often by several orders of magnitude. As a result, for most practical applications, the equation can be approximated by neglecting the *Resonant-term*, leading to the simplified expression given in equation S6. Since the cross-term arises from the interaction between the SAXS and resonant contributions, the resonant term can also be estimated directly from the nonzero *Cross-term*, as shown in equation S7. Without any approximations, however, the scattering terms must satisfy a constraint derived from the Cauchy-Schwarz inequality, which establishes a lower bound for the resonant term when the *SAXS* and *cross-terms* are known, as expressed in equation S8. This constraint is particularly useful in ensuring consistency in data analysis. Both constraints from equations S7 and S8 were applied to energy-dependent simulated data from the spherical model, as described in the main text, to extract the scattering terms. The data processing was performed using the *XAnoS_Components* package within the *XAnoS* software suite, developed at NSF's ChemMatCARS.[6]

$$I(Q,E) \approx I_S + 2f'(E)I_C \quad \text{(S6)}$$

$$I_R(Q) = \langle \rho_r^2(Q)\rangle = \frac{I_C^2(Q)}{I_S(Q)} \quad \text{(S7)}$$

$$I_R(Q) \geq \frac{I_C^2(Q)}{I_S(Q)} \quad \text{(S8)}$$

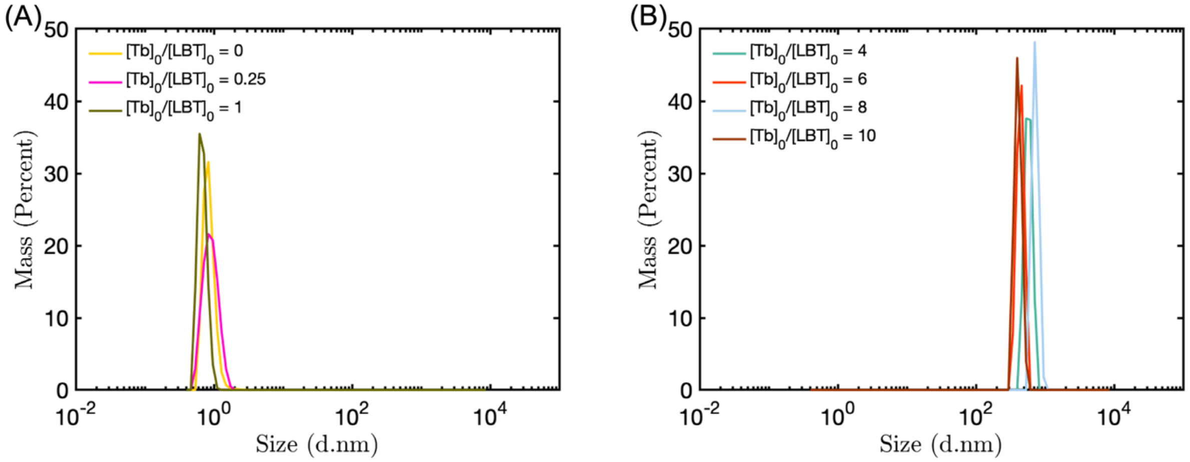

Figure S1: Number size distributions derived from the intensity distribution using Mie theory for solutions containing 100 μM of $LBTLLA^{5-}$ and Tb^{3+} cations at $[Tb^{3+}]_0/[LBT]_0$ ratios of (A) 0, 0.25, and 1: and (B) 4, 6, 8, and 10.

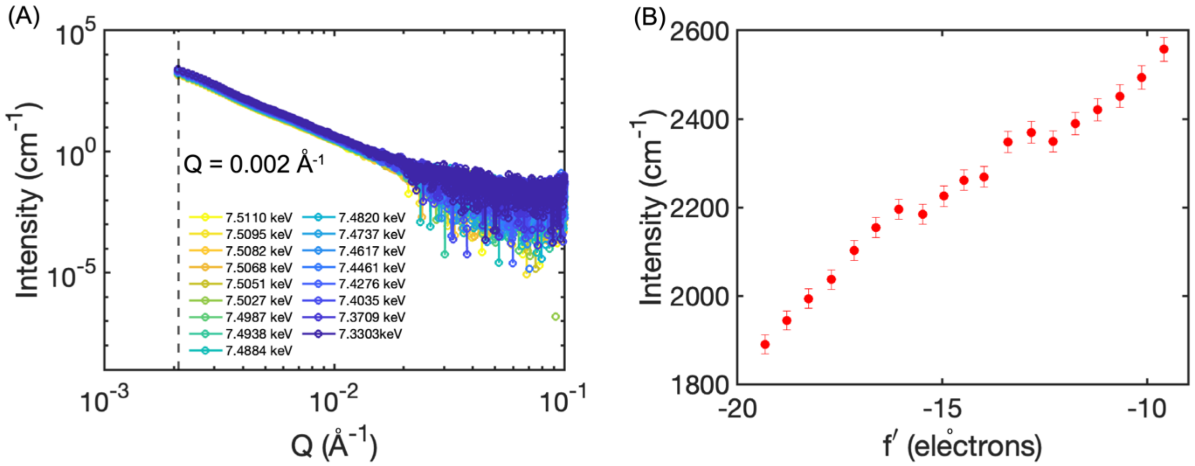

Figure S2: (A) Energy-dependent scattering intensity near the Tb^{3+} absorption edge for aggregates in a solution containing 800 μM LBTLLA and 3.2 mM Tb^{3+}. (B) Corresponding as a function of the real part of the anomalous scattering factor (f') at Q = 0.0021 $Å^{-1}$.

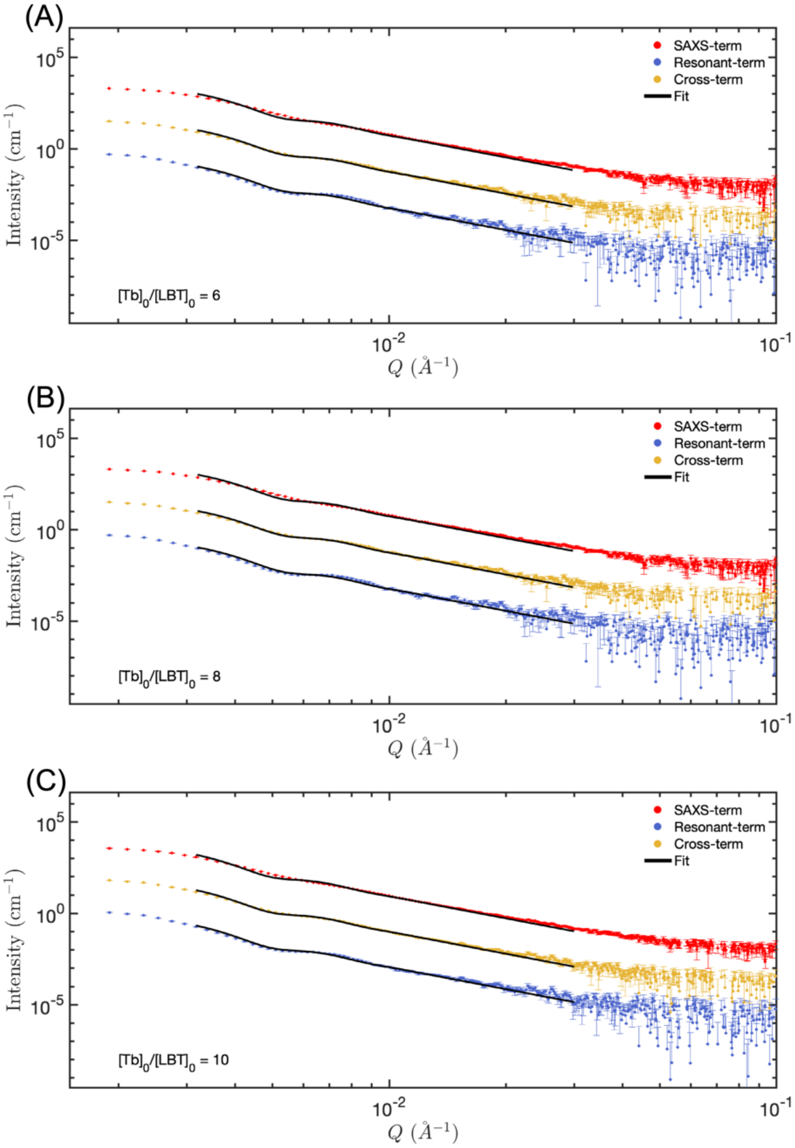

Figure S3: Representative ASAXS profiles and the corresponding fits for a biphasic spherical model from solutions containing Tb^{3+} and 800 μM of $LBTLLA^{5-}$ for ratios (A) $[Tb^{3+}]_0/[LBT]_0 = 6$, (B) $[Tb^{3+}]_0/[LBT]_0 = 8$, and (C) $[Tb^{3+}]_0/[LBT]_0 = 10$.

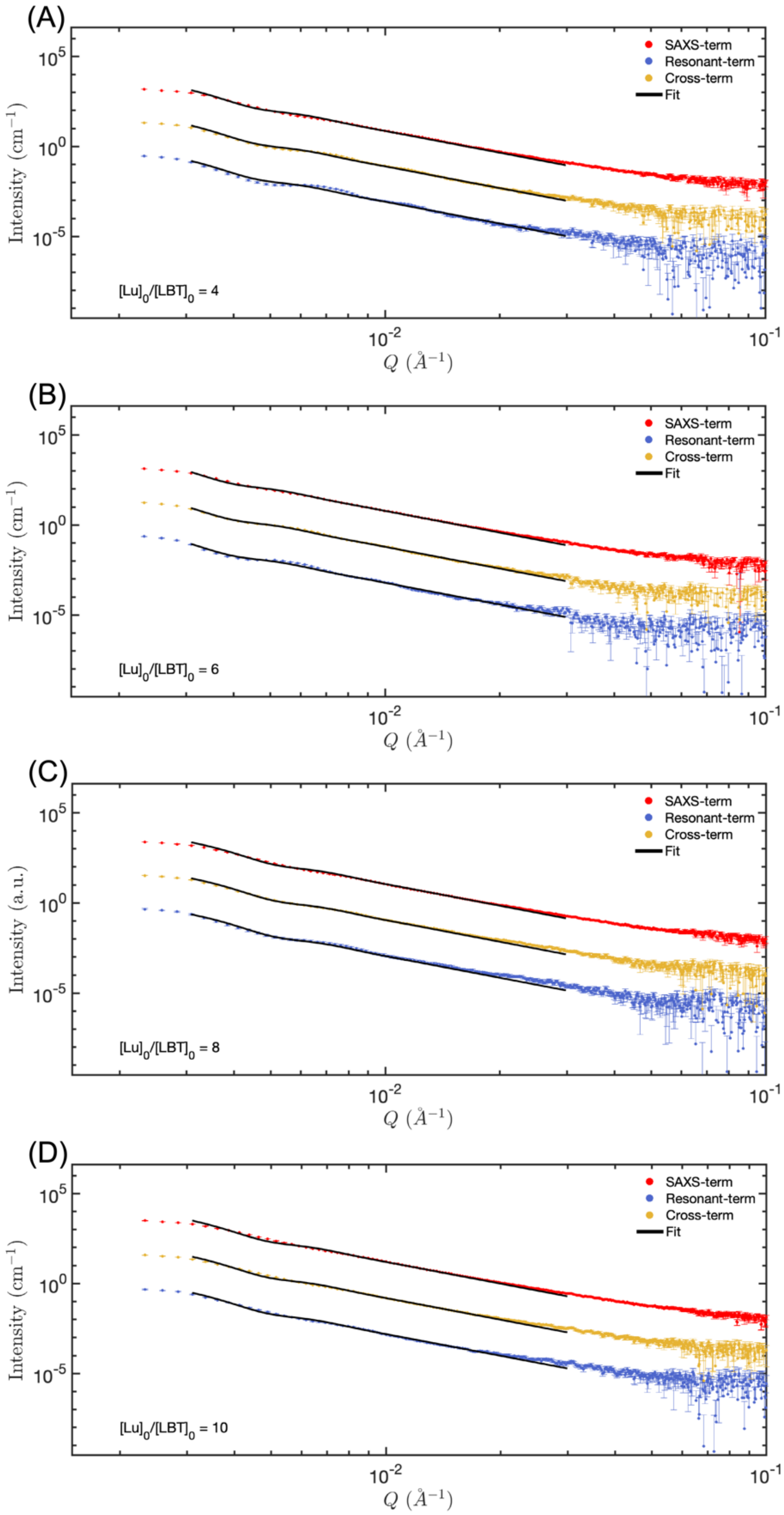

Figure S4: Representative ASAXS profiles and the corresponding fits for a biphasic spherical model from solutions containing Lu^{3+} and 800 μM of $LBTLLA^{5-}$ for ratios (A) $[Lu^{3+}]_0/[LBT]_0 = 4$, (B) $[Lu^{3+}]_0/[LBT]_0 = 6$, (C) $[Lu^{3+}]_0/[LBT]_0 = 8$, and (C) $[Lu^{3+}]_0/[LBT]_0 = 10$.

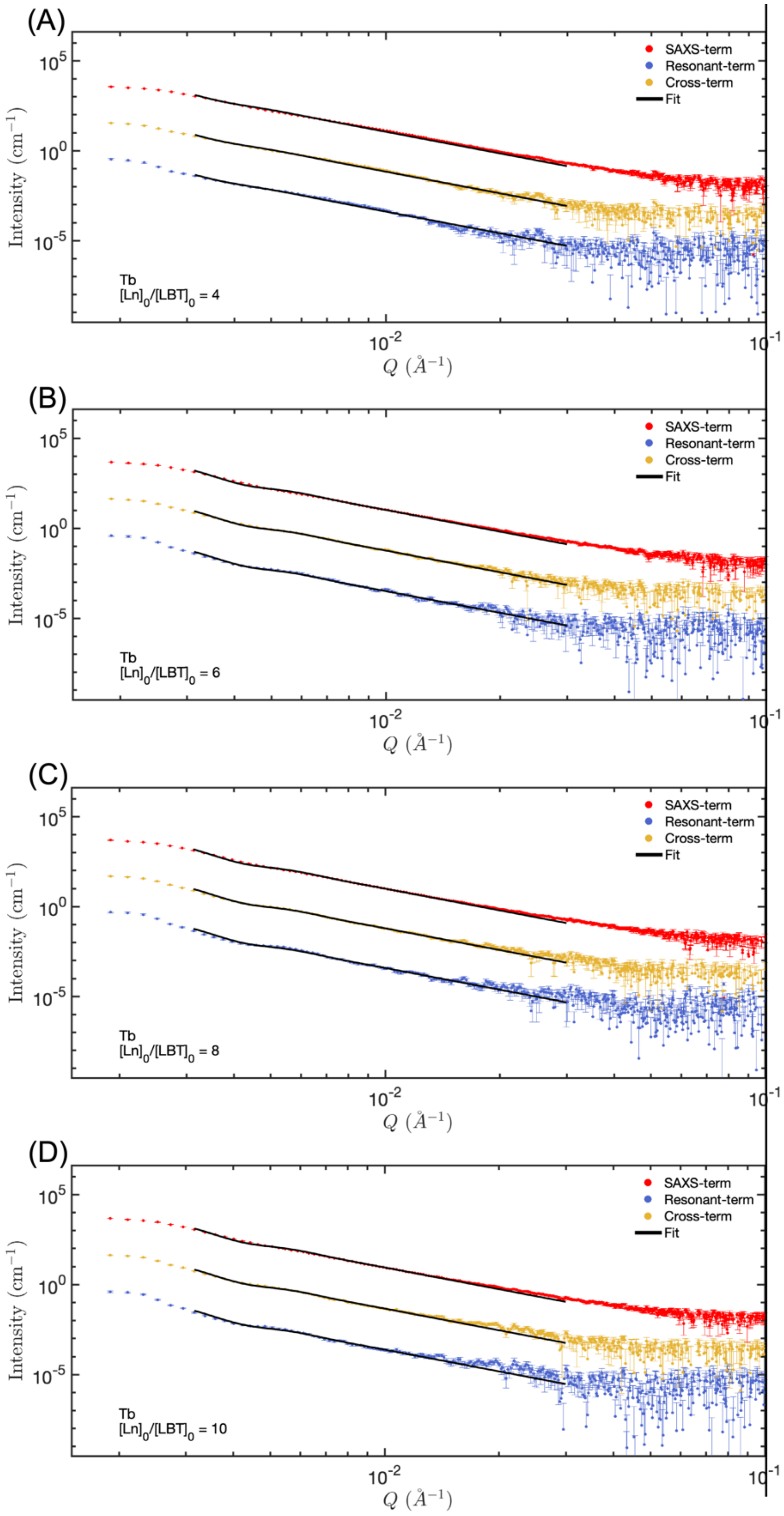

Figure S5: Representative ASAXS profiles (Tb as resonant element) and the corresponding fits for a biphasic spherical model from solutions containing equimolar concentrations of Tb^{3+} and Lu^{3+}, and 800 μM of $LBTLLA^{5-}$ for ratios (A) $[Ln^{3+}]_0/[LBT1]_0$ = 4, (B) $[Ln^{3+}]_0/[LBT]_0$ = 6, (C) $[Ln^{3+}]_0/[LBT]_0$ = 8, and (C) $[Ln^{3+}]_0/[LBT]_0$ = 10.

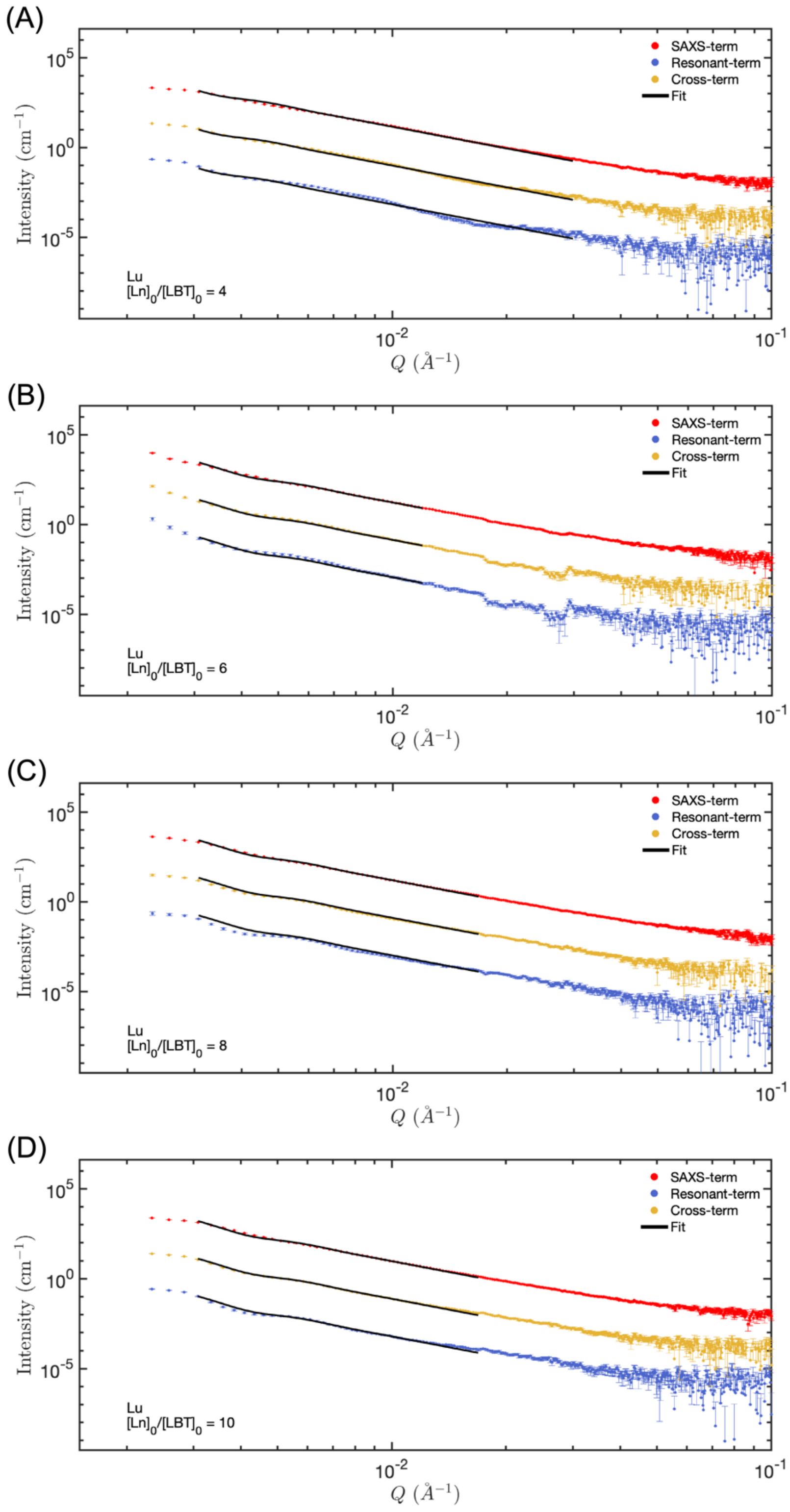

Figure S6: Representative ASAXS profiles (Lu as resonant element) and the corresponding fits for a biphasic spherical model from solutions containing equimolar concentrations of Tb^{3+} and Lu^{3+}, and 800 μM of $LBTLLA^{5-}$ for ratios (A) $[Ln^{3+}]_0/[LBT]_0 = 4$, (B) $[Ln^{3+}]_0/[LBT]_0 = 6$, (C) $[Ln^{3+}]_0/[LBT]_0 = 8$, and (C) $[Ln^{3+}]_0/[LBT]_0 = 10$.

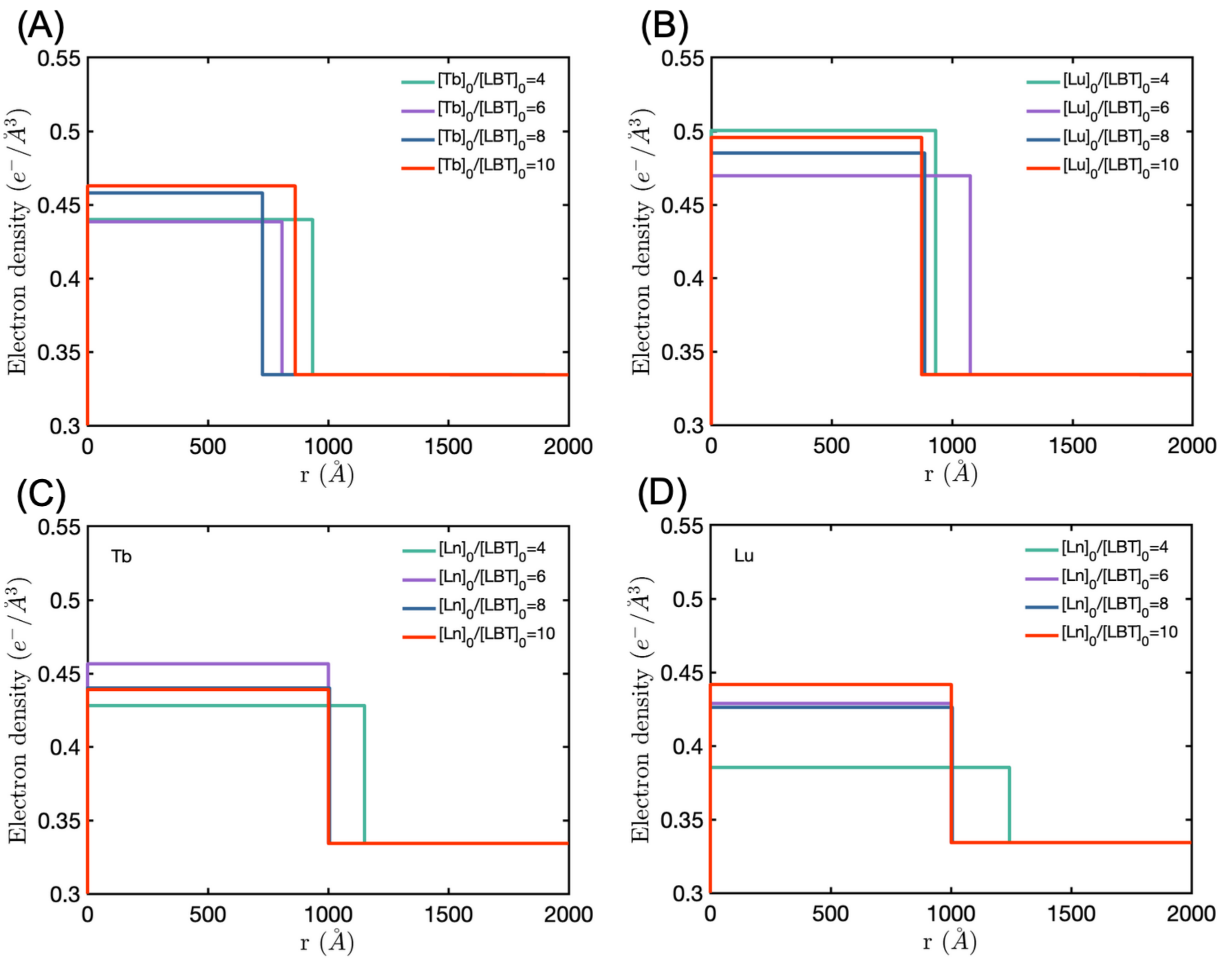

Figure S7. Electron density profiles (total electron density as a function of the radius of spherical structures, r) for different trivalent cations and different $[Ln^{3+}]_0/[LBT]_0$ (A) 800 μM of $LBTLLA^{5-}$ and Tb^{3+} cations, (B) 800 μM of $LBTLLA^{5-}$ and Lu^{3+} cations, (C) 800 μM of $LBTLLA^{5-}$ and equimolar concentration of Tb^{3+} and Lu^{3+} cations (Tb as resonant element), and (D) 800 μM of $LBTLLA^{5-}$ and equimolar concentration of Tb^{3+} and Lu^{3+} cations (Lu as resonant element).

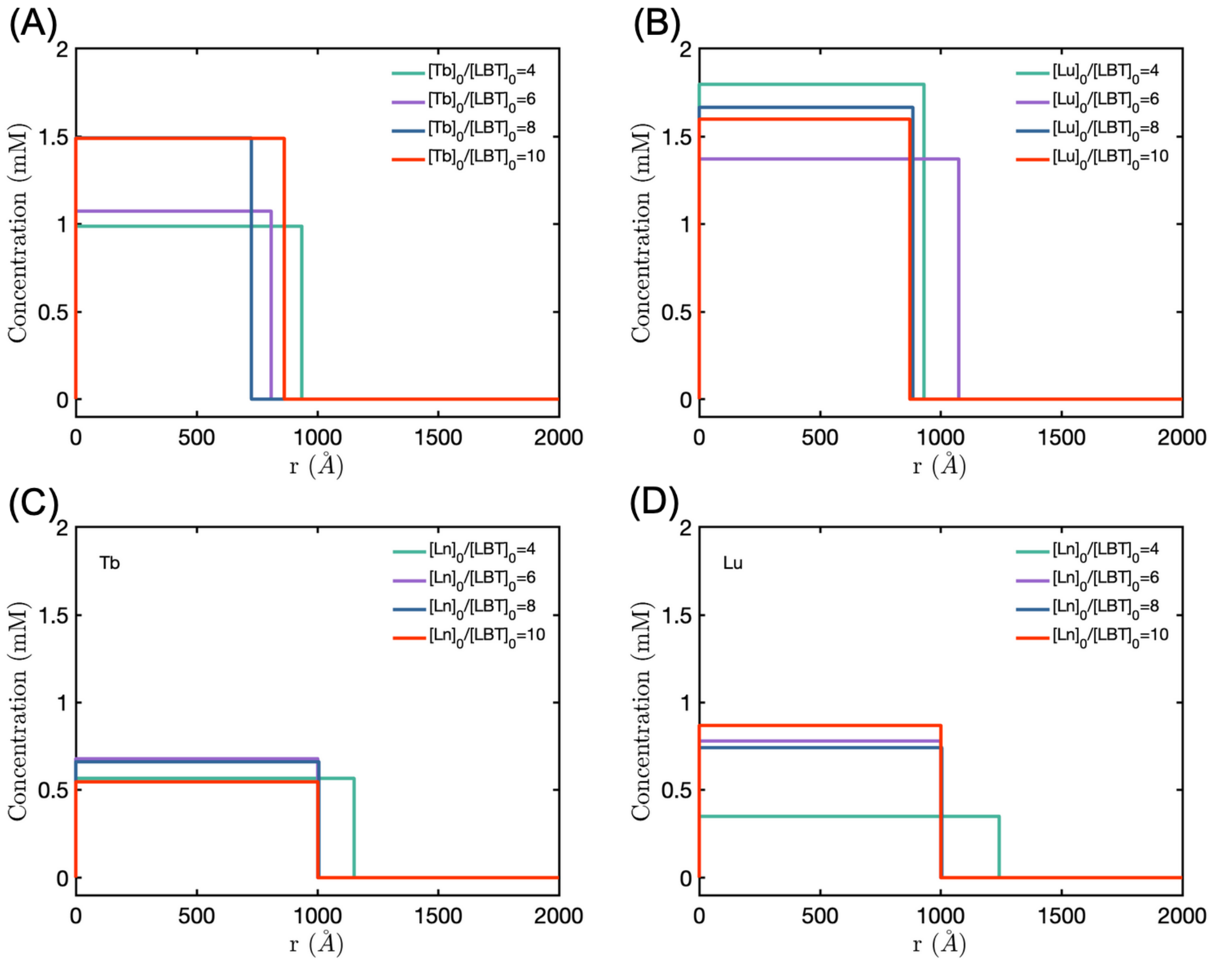

Figure S8. Concentration profiles (concentration of Ln^{3+} cations as a function of the radius of spherical structures, r) for different trivalent cations and different $[Ln^{3+}]_0/[LBT]_0$ (A) 800 μM of $LBTLLA^{5-}$ and Tb^{3+} cations, (B) 800 μM of $LBTLLA^{5-}$ and Lu^{3+} cations, (C) 800 μM of $LBTLLA^{5-}$ and equimolar concentration of Tb^{3+} and Lu^{3+} cations (Tb as resonant element), and (D) 800 μM of $LBTLLA^{5-}$ and equimolar concentration of Tb^{3+} and Lu^{3+} cations (Lu as resonant element).

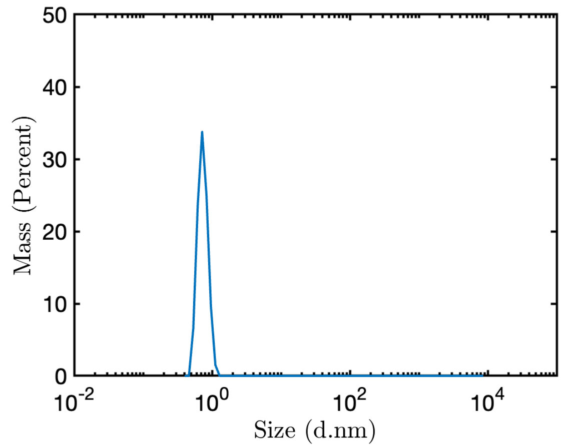

Figure S9: Number-based size distribution calculated from the intensity distribution using Mie theory for the re-dispersed pellet obtained from a solution containing 800 μM of $LBTLLA^{5-}$ and 3.2 mM of Tb^{3+}.

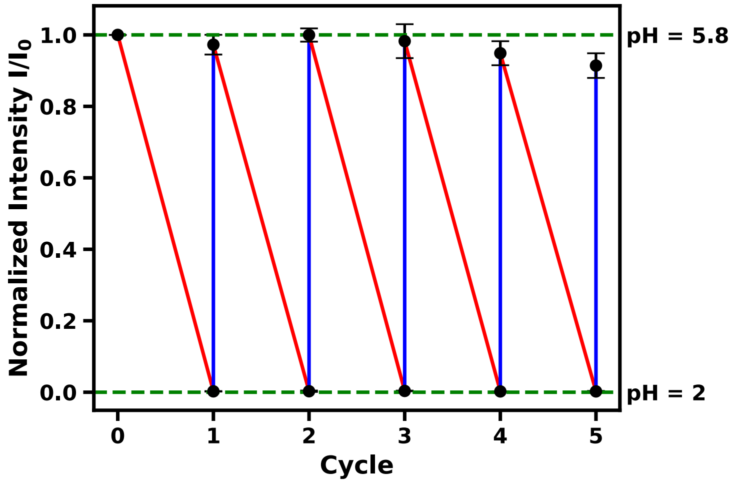

Figure S10: Normalized fluorescence intensity detected at 545 nm (I/I_0) as a function of stripping–rebinding cycles for peptide (100 μM) and Tb^{3+} (400 μM). I_0 represents the initial fluorescence intensity before the first cycle, and I corresponds to the intensity measured after each stripping or rebinding step. Error bars represent the standard deviation from three independent measurements.

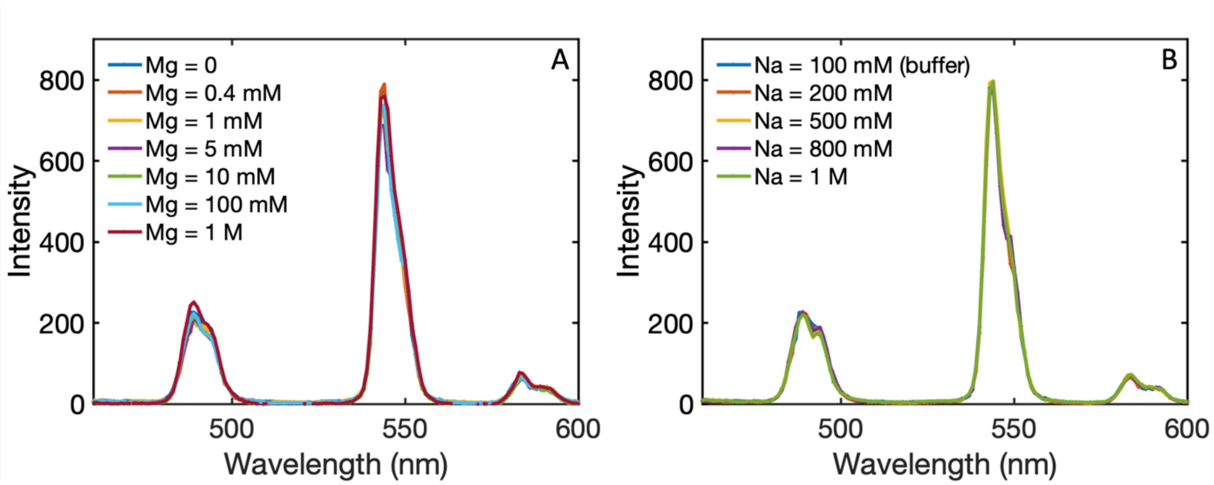

Figure S11: Fluorescence intensity of LBTLLA^{5-} (100 μM) in the presence of Tb^{3+} (400 μM) with increasing concentrations of (A) Na^+ and (B) Mg^{2+}. Samples were excited at 280 nm, and emission was monitored at a wavelength range from 460 nm to 600 nm.

Table S1: Fitting parameters from ASAXS measurements for solutions containing Tb^{3+} and 800 μM of LBTLLA^{5-} at different $[Tb^{3+}]_0/[LBT]_0$ ratios.

Tb/LBT	*Phase1_Density* (g/cm^3)	*Phase1_volFrac*	*Phase1_Rmoles*	*Phase2_Density* (g/cm^3)	*Phase2_volFrac*	*Phase1_R* (Å)	*norm* (nM)
4	1.45±0.03	0.37±0.02	2.0±0.1	0.96±0.08	0.128±0.005	936±2	2.1±0.1
6	1.46±0.02	0.28±0.03	2.3±0.1	1.00±0.10	0.140±0.004	809±2	2.0±0.1
8	1.46±0.03	0.28±0.01	2.2±0.1	0.98±0.05	0.227±0.009	727±1	1.9±0.1
10	1.44±0.02	0.29±0.02	2.3±0.1	1.10±0.10	0.206±0.006	864±2	1.8±0.1

Rmoles is the number of Ln^{3+} per peptide within the self-assembling structures, while norm is the density of these structures. Error bars are determined by mapping the chi-squared space.

Table S2: Fitting parameters from ASAXS measurements for solutions containing Lu^{3+} and 800 μM of LBTLLA^{5-} at different $[Lu^{3+}]_0/[LBT]_0$ ratios.

Lu/LBT	*Phase1_Density* (g/cm^3)	*Phase1_volFrac*	*Phase1_Rmoles*	*Phase2_Density* (g/cm^3)	*Phase2_volFrac*	*Phase1_R* (Å)	*norm* (nM)
4	1.42±0.03	0.35±0.05	2.4±0.1	1.10±0.10	0.350±0.050	931±2	0.76±0.05
6	1.45±0.02	0.33±0.04	2.2±0.1	1.00±0.10	0.193±0.002	1076±1	0.73±0.08
8	1.34±0.02	0.41±0.01	2.2±0.1	1.07±0.03	0.227±0.007	893±12	1.63±0.07
10	1.16±0.02	0.50±0.10	2.0±0.1	1.50±0.20	0.057±0003	1725±63	2.06±0.08

Rmoles is the number of Ln^{3+} per peptide within the self-assembling structures, while norm is the density of these structures. Error bars are determined by mapping the chi-squared space.

Table S3. Fitting parameters from ASAXS measurements (Tb as resonant element) for solutions containing equimolar concentrations of Tb^{3+} and Lu^{3+}, and 800 μM of $LBTLLA^{5-}$ at different $[Ln^{3+}]_0/[LBT]_0$ ratios.

Ln/LBT	$Phase1\_Density$ (g/cm^3)	$Phase1\_volFrac$	$Phase1\_Rmoles$	$Phase2\_Density$ (g/cm^3)	$Phase2\_volFrac$	$Phase1\_R$ (Å)	$norm$ (nM)
4	1.55±0.03	0.50±0.02	1.4±0.1	0.44±0.02	0.110±0.020	1151±2	2.7±0.1
6	1.45±0.02	0.54±0.03	1.2±0.1	0.80±0.10	0.066±0.004	1001±2	2.9±0.2
8	1.44±0.01	0.48±0.02	1.0±0.1	1.19±0.09	0.071±0.001	1005±1	2.2±0.2
10	1.45±0.02	0.54±0.02	1.1±0.1	1.01±0.09	0.053±0.003	1002±1	2.1±0.2

$Rmoles$ is the number of Ln^{3+} per peptide within the self-assembling structures, while $norm$ is the density of these structures. Error bars are determined by mapping the chi-squared space.

Table S4. Fitting parameters from ASAXS measurements (Lu as resonant element) for solutions containing equimolar concentrations of Tb^{3+} and Lu^{3+}, and 800 μM of $LBTLLA^{5-}$ at different $[Ln^{3+}]_0/[LBT]_0$ ratios.

Ln/LBT	$Phase1\_Density$ (g/cm^3)	$Phase1\_volFrac$	$Phase1\_Rmoles$	$Phase2\_Density$ (g/cm^3)	$Phase2\_volFrac$	$Phase1\_R$ (Å)	$norm$ (nM)
4	1.47±0.01	0.237±0.008	0.84±0.06	0.76±0.03	0.062±0.002	1246±2	2.0±0.2
6	1.54±0.03	0.450±0.020	1.00±0.10	0.70±0.02	0.150±0.004	1060±4	2.7±0.2
8	1.49±0.02	0.450±0.020	0.93±0.08	0.62±0.05	0.131±0.003	1063±2	2.2±0.2
10	1.44±0.01	0.500±0.020	1.00±0.10	0.74±0.02	0.159±0.005	1045±1	2.1±0.2

$Rmoles$ is the number of Ln^{3+} per peptide within the self-assembling structures, while $norm$ is the density of these structures. Error bars are determined by mapping the chi-squared space.

References

(1) Ortuno Macias, L. E. et al. Lanthanide binding peptide surfactants at air-aqueous interfaces for interfacial separation of rare earth elements. *Proc Natl Acad Sci USA* **2024**, 121, e2411763121

(2) Paul, H. R.; Bera, M. K.; Macke, N.; Rowan, S. J; Tirrell, M. V. Quantitative Determination of Metal Ion Adsorption on Cellulose Nanocrystals Surfaces. *ACS nano*. **2024** *18*, 1921-1930.

(3) Tatchev, D. Structure analysis of multiphase systems by anomalous small-angle X-ray scattering. *Philosophical Magazine* **2008**, 88, 1751-1772.

(4) Sztucki, M.; Di Cola, E.; Narayanan, T. Instrumental developments for anomalous small-angle X-ray scattering from soft matter systems. *Journal of Applied Crystallography* **2010**, 43, 1479-1487.

(5) Chantler, C. T. Detailed tabulation of atomic form factors, photoelectric absorption and scattering cross section, and mass attenuation coefficients in the vicinity of absorption edges in the soft X-ray (Z= 30–36, Z= 60–89, E= 0.1 keV–10 keV), addressing convergence issues of earlier work. *Journal of Physical and Chemical Reference Data* **2000**, 29, 597-1056.

(6) Bera, M. K; Bu, W. XmodFit: X-ray Modeling and Fitting. https://github.com/chemmatcars/XmodFit (last accessed 2025-01-24).
